# Invertebrate neurophylogeny: suggested terms and definitions for a neuroanatomical glossary

**DOI:** 10.1186/1742-9994-7-29

**Published:** 2010-11-09

**Authors:** Stefan Richter, Rudi Loesel, Günter Purschke, Andreas Schmidt-Rhaesa, Gerhard Scholtz, Thomas Stach, Lars Vogt, Andreas Wanninger, Georg Brenneis, Carmen Döring, Simone Faller, Martin Fritsch, Peter Grobe, Carsten M Heuer, Sabrina Kaul, Ole S Møller, Carsten HG Müller, Verena Rieger, Birgen H Rothe, Martin EJ Stegner, Steffen Harzsch

**Affiliations:** 1 Universität Rostock, Institut für Biowissenschaften, Abteilung für Allgemeine und Spezielle Zoologie, Universitätsplatz 2, D-18055 Rostock, Germany; 2 RWTH Aachen, Institute of Biology II, Department of Developmental Biology and Morphology of Animals, Mies-van-der-Rohe-Straße 15, D-52056 Aachen, Germany; 3 Universität Osnabrück, Fachbereich Biologie/Chemie, AG Zoologie, Barbarastraße 11,, D-49069 Osnabrück, Germany; 4 Biozentrum Grindel/Zoological Museum, Martin-Luther-King-Platz 3, D-20146 Hamburg, Germany; 5 Humboldt-Universität zu Berlin, Institut für Biologie - Vergleichende Zoologie, Philippstraße 13, D-10115 Berlin, Germany; 6 Freie Universität Berlin, Zoologie - Systematik und Evolutionsforschung, Königin-Luise-Straße 1-3, D-14195 Berlin, Germany; 7 Universität Bonn, Institut für Evolutionsbiologie und Ökologie, An der Immenburg 1, D-53121 Bonn, Germany; 8 University of Copenhagen, Department of Biology, Research Group for Comparative Zoology, Universitetsparken 15, DK-2100 Copenhagen, Denmark; 9 Ernst-Moritz-Arndt-Universität Greifswald, Zoologisches Institut, Cytologie und Evolutionsbiologie, Johann-Sebastian-Bach-Straße 11/12, D-17487 Greifswald, Germany

## Abstract

**Background:**

Invertebrate nervous systems are highly disparate between different taxa. This is reflected in the terminology used to describe them, which is very rich and often confusing. Even very general terms such as 'brain', 'nerve', and 'eye' have been used in various ways in the different animal groups, but no consensus on the exact meaning exists. This impedes our understanding of the architecture of the invertebrate nervous system in general and of evolutionary transformations of nervous system characters between different taxa.

**Results:**

We provide a glossary of invertebrate neuroanatomical terms with a precise and consistent terminology, taxon-independent and free of homology assumptions. This terminology is intended to form a basis for new morphological descriptions. A total of 47 terms are defined. Each entry consists of a definition, discouraged terms, and a background/comment section.

**Conclusions:**

The use of our revised neuroanatomical terminology in any new descriptions of the anatomy of invertebrate nervous systems will improve the comparability of this organ system and its substructures between the various taxa, and finally even lead to better and more robust homology hypotheses.

## Introduction

The nervous system is a major organ system in almost all metazoans, with sponges and placozoans the only exceptions. Its fascination comes from its complexity, particularly in vertebrates, and its enormous diversity in invertebrates. The first detailed descriptions of invertebrate nervous systems were published over 150 years ago, and the evolution of nervous systems of all kinds has been the focus of evolutionary morphologists for many decades. Particularly noteworthy in this regard are the Swedish neuroanatomists N. Holmgren (1877-1954) and B. Hanström (1891-1969). Their comparative research across a broad range of invertebrate taxa contributed immensely to our knowledge of nervous system architecture. Hanström was also the first scientist to reconstruct phylogenetic relationships in detail on the exclusive basis of neuroanatomical characters, a tradition which was continued by Sandeman et al. [1] and Strausfeld [2], among others, using cladistic approaches. The more general combination of a detailed analysis of neuroanatomical characters followed by their interpretation in a phylogenetic and evolutionary context was christened 'neurophylogeny' by the Canadian neurobiologist Dorothy Paul [3,4], a term made popular by Harzsch [5,6]. The renaissance of 'neurophylogeny' in the last two decades has been fuelled by immunohistochemistry and confocal-laser-scanning microscopy, techniques which have revolutionized the study of nervous systems. In combination, these techniques allow nervous system structures to be documented much more intuitively than was ever previously possible using serial sections and TEM, and, equally importantly, in a much higher number of species. In addition to the architecture of the nervous system it has also become possible to study the expression of certain neurotransmitters, which in turn makes it easier to identify specific structures (e.g., individual neurons). These new techniques have encouraged many zoologists to re-investigate the nervous system of various animal taxa and to explore it in taxa in which it had not been studied previously. Many of these studies have provided detailed structural analyses in the framework of what has been called 'New animal phylogeny' [7].

Decades of detailed descriptions combined with the diversity of nervous systems, which range from the relatively simple neural architectures in groups such as cnidarians and platyhelminthes to the highly complex nervous systems in insects and cephalopods, have, however, resulted in a wealth of neuroanatomical terms which it is almost impossible to keep track of. The terminology covers all levels of the structural hierarchy. On the highest level, the nervous system as a whole has, for example, been described as either a 'plexus', an 'orthogon' or a 'rope-ladder-like nervous system' representing alternative types of organizations. On a lower level, specific subunits of nervous systems, such as the 'central body' and the 'protocerebral bridge' in the arthropod brain, have also been identified. On the cellular level, cell biologists have built up a detailed terminology of nerve and receptor cells. However, many terms, even very general ones such as 'brain', 'nerve', and 'eye', are used in varying ways in the different animal groups, and no consensus on their exact meaning exists. Not only are terms used differently in different taxa, varying research interests have also brought forth their own terminology, with the most significant differences being between the nomenclature used by physiologists and functional morphologists on the one hand and that preferred by comparative and evolutionary morphologists on the other. For most features of the nervous system, knowledge about their function and physiology extends right down to the molecular level. Strictly speaking however, this only holds true for a very limited number of organisms, primarily vertebrates and hexapods and a few other taxa. As a result, morphologists often need to draw inferences about the function of certain structures by analogy. If we intend to use a morphological terminology which covers all the metazoans, it should, therefore, preferably be based on structure and topology rather than function [8]. This ties in with our main objective, which is to trace the evolution of the morphology of the nervous system in invertebrates on the basis of the evolutionary transformations implied by their phylogenetic relationships.

Recently, a general debate has arisen over how a higher degree of transparency, inter-subjectivity, reproducibility and communicability can be obtained when it comes to morphological data. Although it is generally agreed that a more precise, standardized terminology will be necessary in the future [9-13], varying proposals have been made with regard to what it should be based on. It has been suggested on the one hand that morphological descriptions should be independent of any homology assumptions [8,11], while on the other, primary homologies have explicitly been put forward as the basis for a "morphological terminology" [10]. In our view, in the comparative framework of phylogenetic analyses, the two approaches complement each other. We agree that morphological descriptions and terminology should be free of any assumptions regarding homology, and not be restricted to certain taxa. However, if, as parts of organ systems, structures are conceptualized as character states and characters for the purposes of phylogenetic analysis, primary homology is necessarily implied (e.g., [8,14]). Applying a specific term to a character state (or character) after a test of primary homology (e.g., [15-17]) implies that the state and the character are homologous.

We all need to be aware that after 150 years of research into evolutionary morphology, every single morphological description and term used is affected by an evolutionary interpretation of the morphology and structures in question. Often, terms do not even refer to exact descriptions but imply some kind of generalization, revealing that typological thinking is still present in our terminology. Morphological terminology is not a pristine field, and it is important that we take this into account in our dealings with it.

Fully aware of the problems of such an approach, we herein provide a glossary which we suggest be used as a guide through the field of neurophylogeny and taken as a starting point in formulating definitions of characters and character states in phylogenetic character matrices. For each term, extensive background is provided, outlining the history of the term and explaining how it has already played a role in the discussion of nervous system evolution. In addition, we discourage certain other terms which are either synonymous with the favoured term or whose relationship to the favored term is unclear. We advocate the use of precise and consistent terminology which is taxon-independent and free of homology assumptions, but the long tradition of descriptive nervous system morphology has not been ignored in the making of this glossary and the general and established use of any single term has thus been taken into consideration. Taxon-independence does not cancel out the fact that the greater the detail in which a term is defined, the more its application will be restricted to certain taxa. Many general features are defined on the basis of the seminal account by Bullock and Horridge [18], but almost 50 years later it has often been necessary to update the terminology used by those authors. We hope that the use of our revised neuroanatomical terminology in any new descriptions of the anatomy of invertebrate nervous systems will improve the comparability of this organ system and its substructures between the various taxa, and finally even lead to better and more robust homology hypotheses.

We restrict our glossary mainly to general neuroanatomical terms that are applicable to all or almost all invertebrate taxa, but do include more specific terms in several groups. We also include terms for sensory organs, particularly light-sensitive organs. We have chosen those terms which, to our knowledge, have the greatest impact on the discussion of the evolution of nervous systems. It goes without saying that the restrictions we have imposed also reflect the expertise of the authors of the present glossary. The format defined herein will facilitate the addition of new entries in the future.

Our suggestions for a glossary come at a time when formalized 'ontologies' - defined and controlled vocabularies which are computer interpretable - are beginning to play a role (e.g., [19-24]). These will undoubtedly be an important tool in all future morphological work [9,11,12], and our definitions try to take this into account by following a specific formalized scheme and, in particular, by explicitly indicating class-subclass and part-whole relationships. Neuroanatomical ontologies are already very popular in biomedicine (e.g., [25-30]), and although most ontology projects in zoology have so far focused on single model system species (e.g., *Drosophila melanogaster, Caenorhabditis elegans*) or morphologically relatively well-defined taxa (e.g., Hymenoptera, Amphibia), the field is growing rapidly (see NCBO BioPortal: http://bioportal.bioontology.org/ for projects which are planned or underway). Developing anatomy ontologies for the entire group of metazoans or at least all invertebrates will be a much greater challenge and a goal that will occupy research groups all over the world for many decades (for initial attempts see the Common Anatomy Reference Ontology, CARO [31], and UBERON, http://obofoundry.org/wiki/index.php/UBERON:Main_Page).

**Figure 1:**
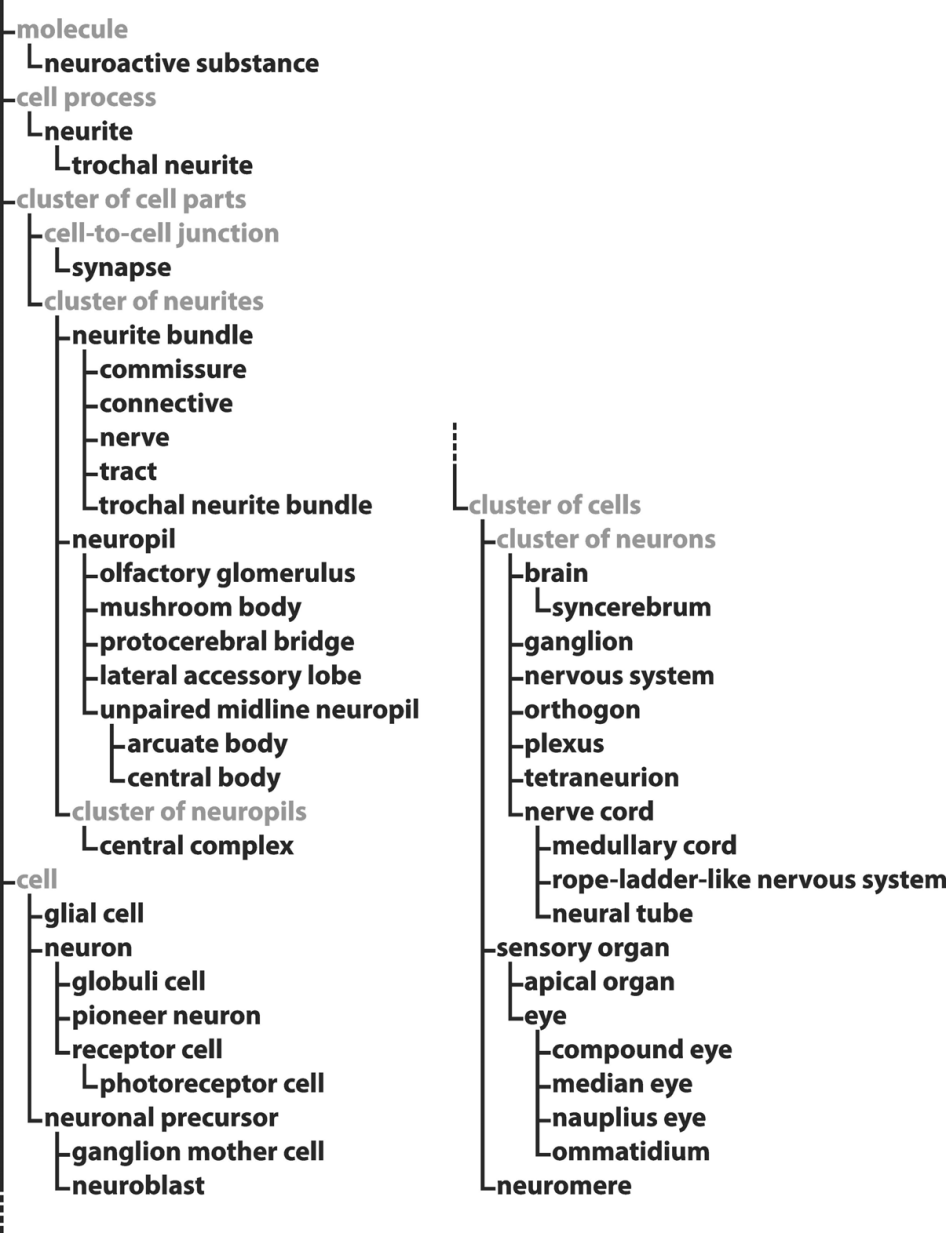
**Taxonomic ontology of all 47 terms defined in this work (printed in black)**. Only class-subclass relationships are shown.

All the definitions in this glossary are organized according to Aristotelian definitions (definitions *per genus et differentiam *see, e.g., [11,12,20,32]). Each definition is composed of two parts: (i) The *genus *part specifies which general (parent) term this (child) term is a more specific subtype of. This results in a hierarchy of more and more inclusive terms which is based on class-subclass relationships (Figure 1; see also taxonomic inclusion, [33]). This hierarchy is generally referred to as a taxonomy (i.e., *taxonomy *in a broad sense). Within taxonomies, the defining properties are inherited downstream (downward propagation) from a given class to all its subclasses. Therefore, the *genus *part of a definition functions like a shorthand and stands for the defining properties of all the term's parent terms. It specifies the set of properties that each instance of the defined type *necessarily *possesses, though possession of these properties is not, in itself, *sufficient *for the instantiation of the type. (ii) The *differentia *part, on the other hand, specifies the set of properties that distinguish the type to be defined from all the other sub-types of the parent type. The combination of *genus *and *differentia *specifies the set of properties that is *sufficient *for the instantiation of the defined type. As a consequence, the *genus *and the *differentia *part of a given term's definition together provide the *genus *part of all of its direct subsidiary terms.

The definitions in this glossary are organized according to the following scheme, with the first sentence representing the *genus *part and all subsequent sentences the *differentia *part of the definition:

### **{#} Defined term**

The defined term is a (type of) **➞its parent term**. It is part of a/the **➞other term**. We use 'part of the' in the sense of 'part of every' and 'part of a' in the sense of 'part of some'. Further defining properties may follow.

Those neuroanatomical terms which are printed in bold and with an arrow are **➞main entries**; they were given a specific definition and numbered from {1} to {47}. Those neuroanatomical terms which are printed without an arrow and in bold are **side entries**; they do not have a specific definition but are likewise important for neuroanatomical descriptions. Table 1 lists all main entries and side entries with their positions in the text - this will be a helpful tool when using this glossary.

## Entries

### **{1} Apical organ**

The apical organ is a **➞sensory organ**. It is part of a **➞nervous system **and comprises an apical ciliary tuft and **➞receptor cells**. It is located at the anterior pole of larvae.

**Discouraged terms:** apical ganglion, apical rosette, apical plate

**Figure 2:**
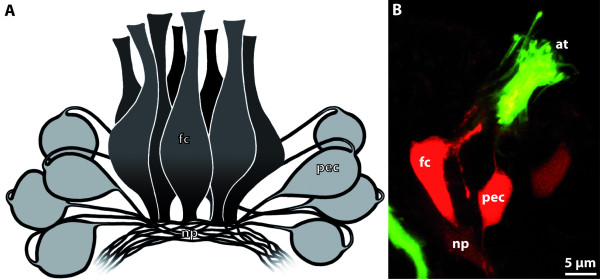
**Apical organ of the creeping-type larva of the entoproct *Loxosomella murmanica***. **A**. Eight bipolar peripheral cells are arranged around eight central flask-shaped receptor cells which are underlain by a central neuropil. [Schematic drawing based on serotonin-like immunoreactivity.] **B**. Flask-shaped receptor cell, situated just below the apical ciliary tuft, and peripheral cell with two emerging neurites. [Confocal laser scanning micrograph showing serotonin-like immunoreactivity.] Abbreviations: at = apical ciliary tuft; fc = flask-shaped receptor cell; np = neuropil; pec = peripheral cell. Originals: A. Wanninger.

**Background/comment: **In most representatives of Lophotrochozoa, the apical organ consists of a specific number of **flask-shaped receptor cells **and displays serotonin-like immunoreactivity (SLI), and sometimes also FMRFamide-like immunoreactivity (RFLI) (Figure 2). Additional cell types such as the ones bearing the cilia that contribute to the apical ciliary tuft are present. The larval apical organ is a major sensory system which often is said to be of importance in detecting settlement cues, though this has never been proven experimentally. Arguments against this notion are the fact that several taxa are known to undergo metamorphosis without having an apical organ (e.g., Echiura [34,35]) or to lose the apical organ prior to the onset of metamorphosis (e.g., in Scaphopoda [36]). Most spiralian larvae have about 4 flask-shaped receptor cells displaying SLI. However, polyplacophoran larvae and creeping-type entoproct larvae differ from the common spiralian phenotype in that they have 8-10 flask-shaped receptor cells and an additional set of peripheral cells, rendering their apical organs the most complex among spiralian larvae (Figure 2). This is considered an apomorphy of a proposed monophyletic Tetraneuralia (**➞tetraneurion**) comprising Entoprocta and Mollusca [37].

In some studies the term 'apical ganglion' has been ascribed to the larval part of the anterior sensory organ of spiralian larvae, which often coexists with the early rudiment of the forming adult **➞brain**. The two structures together, i.e., the larval and the adult components of the anteriormost neural structures in late-stage spiralian larvae, are then sometimes referred to as the 'apical organ' [38]. The use of these terms is misleading both because the larval components usually only comprise a loose assemblage of cells which do not form a distinct **➞ganglion **and because the larval components might be entirely absent, rendering the term 'apical organ' synonymous with brain in these species. Accordingly, the term 'apical ganglion' should be eliminated and 'apical organ' only be applied in accordance with the definition provided above, i.e., to the anterior larval sensory organ that bears flask-shaped receptor cells and gets lost during metamorphosis.

In most lophotrochozoans, the adult brain or so-called **cerebral commissure **forms at the base of the flask-shaped cells of the apical organ prior to the resorption of the latter. This is usually considered to be evidence of the role of the larval apical organ in the induction of the formation of the adult brain in Lophotrochozoa.

### **{2} Arcuate body**

The arcuate body is an **➞unpaired midline neuropil**. It is part of a **➞syncerebrum **and connected to second order visual **➞neuropils **and to postoral neuropils.

**Discouraged terms: **none

**Background/comment: **Strausfeld [39] introduced the term arcuate body to denominate an unpaired midline neuropil in the chelicerate brain that had formerly been called **➞central body **[40]. The neuroanatomical evidence that distinguishes the arcuate body from the central body is mainly provided by its connectivity: unlike the arcuate body, the central body is only indirectly related to sensory neuropils and has no direct projections to postoral neuropils [39,41]. Apart from chelicerates, an unpaired midline neuropil exhibiting a similarly close relationship to the visual system has also been described for the onychophoran species *Euperipatoides rowelli *[42].

### **{3} Brain**

A brain is a cluster of **➞neurons**. It is part of a **➞nervous system**. It is the most prominent anterior condensation of neurons and may also include further types of cells, including **➞glial cells **and **pigment cells**.

**Discouraged terms:** cerebral ganglion, supraesophageal ganglion

**Figure 3:**
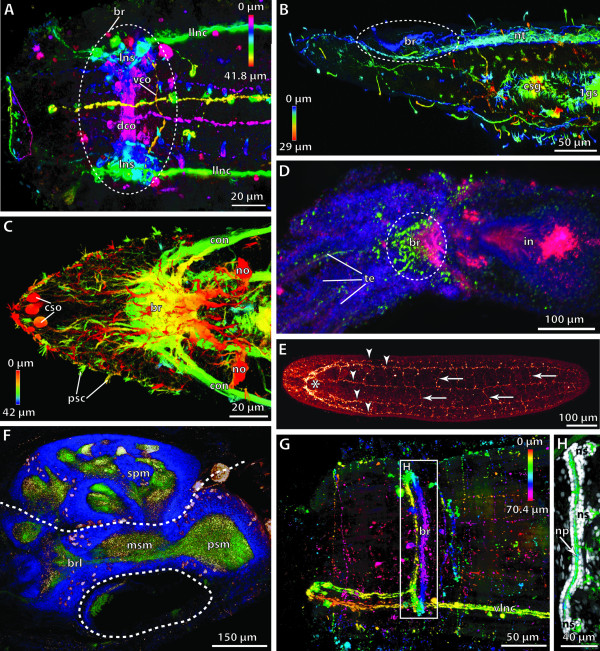
**Different types of brains in invertebrates**. **A**. Commissural brain in *Macrodasys *sp. (Gastrotricha). [FMRF-amide-like immunoreactivity. Depth-coding image.] **B. **Early cephalochordate larva, lateral view. [Acetylated α-tubulin immunoreactivity. Depth-coding image.] **C**. Compact brain in the polychaete *Scoloplos armiger *(Orbiniidae). [Acetylated α-tubulin immunoreactivity. Depth-coding image.] **D. ***Cephalodiscus gracilis *(Pterobranchia), dorsal view. [Serotonin-like (green) and acetylated α-tubulin immunoreactivity (red), and nuclear stain (blue).] **E. **Plexus-like nervous system in the acoel flatworm *Symsagittifera roscoffensis*. [Serotonin-like immunoreactivity.] **F**. Brain of a prehatching embryo of the cephalopod *Octopus vulgaris*. [Acetylated α-tubulin (green) and serotonin-like (red) immunoreactivity, and nuclear stain (blue). Anterior to the left.] **G, H. **Cycloneuralian brain in Priapulida. **G. **Cycloneuralian brain of the larva of *Tubiluchus troglodytes *(rectangle). [Serotonin-like immunoreactivity.] **H**. Brain from the rectangle in G. [Serotonin-like immunoreactivity and nuclear stain (white).] Abbreviations: 1gs = first gill slit; br = brain; brl = brachial lobe; con = circumesophageal connective; csg = club-shaped gland; cso = ciliary photoreceptor-like organ; dco = dorsal commissure; in = intestine; llnc = lateral longitutinal neurite cord; lns = lateral neuronal somata; msm = middle subesophageal mass; no = nuchal organ; np = neuropil; ns = neuronal somata; nt = neural tube; psc = primary sensory cells; psm = posterior subesophageal mass; spm = supraesophageal mass; te = tentacle; vco = ventral commissure; vlnc = ventral longitudinal neurite cord. Originals: A: A. Schmidt-Rhaesa; B, D: T. Stach; C: V. Wilkens, Osnabrück; E: H. Semmler, A. Wanninger; F: T. Wollesen, A. Wanninger; G, H: B.H. Rothe, A. Schmidt-Rhaesa.

**Background/comment: **Adhering to the definition provided above, the criterion of anteriority excludes the use of the term brain in organisms, which do not possess an anterior-posterior body axis. The term is thus not applicable either to the circumoral concentrations of neurons observed in cnidarian polyps or around the manubrium of medusae, or to the thickened **➞neuropil **around the mouth opening of echinoderms. Neither do the neuronal condensations in the **rhopalia **of Cubozoa [43] qualify as brains under this definition. In Phoronida, Brachiopoda and Enteropneusta, a brain is not present after metamorphosis [44]. The position of the brain is usually dorsal of the intestinal system (often the esophagus or pharynx), regardless of whether the attaching **➞nerve cord **is dorsal or ventral (e.g., Figure 3B). This also applies to metazoans with a reduced intestinal system (e.g., Acanthocephala). In a few exceptional cases, such as in the nematomorph *Nectonema*, the brain is ventral of the intestinal system [45].

In some taxa, similar types of brain organization have historically received specific designations (Figure 3). The term **cycloneuralian brain **thus characterizes an organizational mode in which a neuropil of almost uniform thickness surrounds the anterior part of the intestinal system in a ring-like fashion (Figure 3G, H). This is observed in Nematoda, Priapulida, Kinorhyncha and Loricifera [46]. Representatives of Gastrotricha, however, possess a **commissural brain **different from the cycloneuralian brain (Figure 3A; [47]). In some representatives of the Acoela [48,49], only a variable anterior dorsal condensation of neurons is present (Figure 3E). This high variation in the degree of anterior neural concentration suggests that a condensation event occurred independently in the various acoel lineages and that the "uracoel" only had a weakly concentrated nervous system and not a "commissural brain" *sensu *Raikova et al. [48].

Some taxa possess a **compound brain **that is formed by the morphological fusion of embryologically separate ganglion-anlagen. In taxa with segmental body organization, at least some of the subunits constituting the compound brain may have originated in metamerically arranged pairs of **➞ganglia**, as is generally assumed to be the case in arthropods. However, there is ongoing debate about the number and nature of the subunits of the arthropod **➞syncerebrum**. Similarly, the possible segmental origin and subdivision of the annelid brain has also long been a matter of dispute ([50], discussed in [51,52]). In many annelids the brain develops from the larval episphere, whereas the paired ganglia of the trunk segments have their origin in the hyposphere [53]. The adult brain is linked the postoral segmental paired ganglia via circumesophageal **➞connectives **(Figure 3C; [52]). In most annelids, the brain contains specific neuropil compartments and a number of **➞tracts **(e.g., [52,54-56]). Furthermore, distinct commissural ganglia situated on the circumesophageal connectives may be present in certain taxa. Nevertheless, annelid development does not unambiguously support the view that the preoral annelid brain is composed of a number of segmental pairs of ganglia. What there may be is a certain degree of cephalization of the first trunk segments (peristomium and following segments), which often bear sensory appendages instead of regular parapodia (e.g., [57,58]. The ganglia of the corresponding segments are often more or less fused to form a large suboesphageal ganglion. In certain taxa the anteriormost trunk ganglia are closely connected to the preoral brain [54], resulting in a structure that could be considered a 'perioral compound brain' (see [53]). The cephalization of trunk segments renders the posterior boundary of the annelid brain somewhat ambiguous (compare with the situation of the **tritocerebrum **in arthropods, see **➞syncerebrum**).

As in many annelids, the brain in certain Mollusca develops from the larval episphere, whereas the more posterior ganglia (pleural ganglia, pedal ganglia, etc.) arise from ectoderm of the larval hyposphere [44]. The sophisticated brain of Cephalopoda (Figure 3F) exhibits a degree of neural concentration which is exceptional not only among Mollusca but in invertebrates as a whole. This concentration resulted from the fusion of the individual sets of ganglia present in the last common gastropod-cephalopod ancestor. Although the cephalopod brain circumscribes the esophagus, the number, nature and relative position of its parts differ greatly from the condition seen in arthropods. In some - but by no means all - invertebrate brains, regions of neuronal **somata **and **➞neurites **(**➞neuropil**) can be distinguished (see, e.g., [59]). In spiralians, somata usually surround a central neuropil. In most cycloneuralian brains, the somata are anterior and posterior to the neuropil and in the commissural brain of gastrotrichs the somata are lateral to the commissural neuropil [47]. In the deuterostome taxa Pterobranchia and Tunicata we recommend using the term 'brain' for the distinct anterior dorsal clusters of neurons, despite the traditional use of 'ganglion' for these structures (e.g., [60-63]. The brain architecture in Pterobranchia differs considerably from that in Tunicata. The brain of pterobranchs consists of a basiepidermal concentration of **axons **(Figure 3D; [60,63]). It is not known for certain how the neuronal somata in these brains are arranged, but they seem to constitute a **cell cortex **that surrounds a neuropil. In tunicates, the dorsal brain is surrounded by an extracellular matrix [61,62]. Peripheral **➞nerves **originate from the brain, which displays a central neuropil and peripheral somata.

### **{4} Central body**

The central body is an **➞unpaired midline neuropil**. It is a part of the **➞central complex**. It is composed of tangential and columnar **➞neurons**. These neurons form horizontal layers and provide a connection to the **➞lateral accessory lobes **and the **➞protocerebral bridge**. Subpopulations of the columnar neurons cross the midline of the **➞syncerebrum **within the central body or before entering the central body.

**Discouraged terms**: none

**Figure 4:**
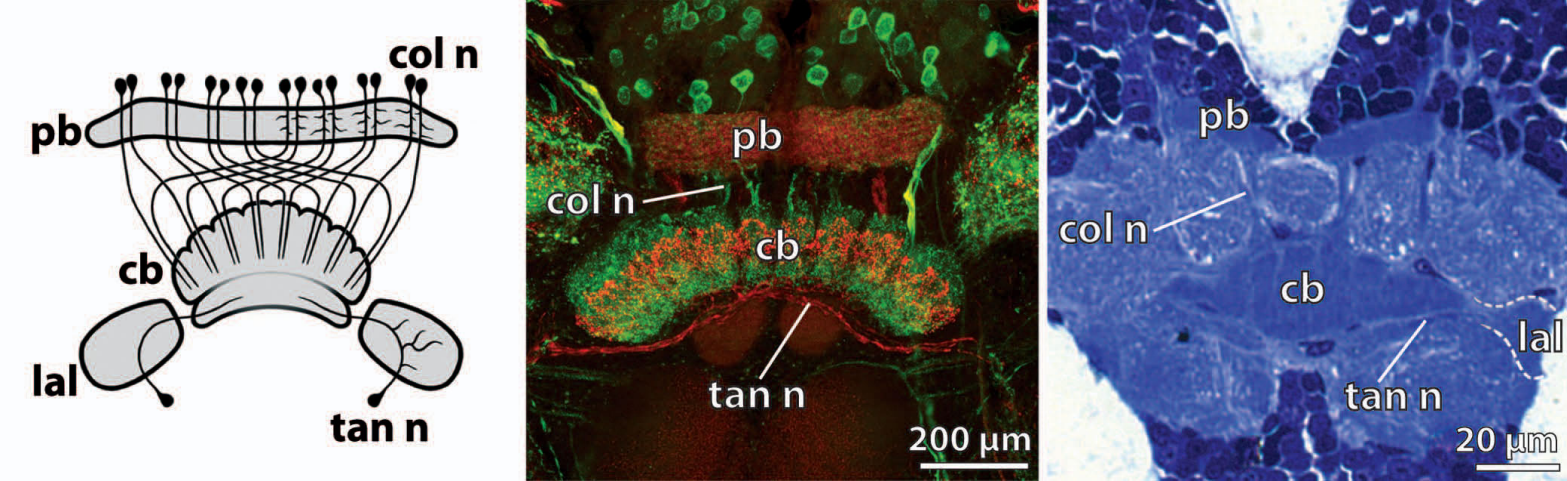
**The central complex assembly**. **A**. Schematic representation of the insect central complex compared to **B**. an original staining of the corresponding neuropils in the brain of the cockroach *Periplaneta americana*. [Frontal section, double-labeling showing allatostatin-like immunoreactivity (red) and tachykinin-like imunoreactivity (green).] **C**. The neuropils of the central complex in the malacostracan *Spelaeogriphus lepidops *correspond to those in insects, but the protocerebral bridge is split. Unlike in insects, the neurite bundles keep ipsilateral between protocerebral bridge and central body. [Frontal semi-thin section. Methylene-blue staining.] Abbreviations: cb = central body; col n = columnar neurons; lal = lateral accessory lobes; pb = protocerebral bridge; tan n = tangential neurons. Originals: A: C.M. Heuer; C: M.E.J. Stegner; B: Modified from [64], with permission of Elsevier.

**Background/comment: **Detailed descriptions of the neuroanatomy of the central body are available for various insects (for a synopsis of the relevant literature see [64]). In this group, the central body consists of two subunits (Figure 4A, B) termed the upper and lower division [65] or, alternatively, the fan-shaped and ellipsoid body [66]. Both terminologies are in use today. The central body in Crustacea also exhibits horizontal layers but lacks a distinct separation into an upper and lower division (Figure 4C). Single unpaired midline neuropils exhibiting central body-like architectural characters have also been described in Myriapoda, Chelicerata and Onychophora [54]. Strausfeld [39,42,67] introduced the term **➞arcuate body **for these taxa. As yet, any attempts to homologize these single unpaired midline neuropils with individual components of the central complex have failed due to the absence of the specific connectivities that define the **➞neuropils **in the central complex.

### **{5} Central complex**

The central complex is a cluster of **➞neuropils**. It is part of a **➞syncerebrum**. It consists of three interconnected subunits: the unpaired **➞central body**, which is situated in the middle of the neuropil assembly, the unpaired **➞protocerebral bridge **and the paired **➞lateral accessory lobes**.

**Discouraged terms**: none

**Background/comment: **Within the framework of the central complex, the central body mediates between the protocerebral bridge and the lateral accessory lobes (Figure 4). All three subunits of the central complex are linked to other parts of the **protocerebrum**. In those species studied in detail, connections between the central complex and the postoral neuropils are established via the lateral accessory lobes. Assemblies of neuropils in the sense of the definition have only been described in Arthropoda to date. Williams [68] contributed significantly to resolving the internal architecture and the connectivity between the protocerebral bridge, central body and lateral accessory lobes. A central complex has been identified in all insect orders investigated so far (Figure 4A, B; [65]). In Crustacea, a central complex adhering to the architectural scheme found in Insecta has been described in representatives of Malacostraca (Figure 4C; [64,69]), Remipedia [70] and Branchiopoda [71]. Although it is generally thought that the components of the central complex are part of the ground pattern of the Tetraconata [39,64,69-72], the absence of at least some components of the central complex in certain crustaceans might well be interpreted as plesiomorphic [73]. Several lines of evidence suggest that the central complex acts as a higher navigational and locomotor control centre [74,75].

### **{6} Commissure**

A commissure is a **➞neurite bundle**. It is part of a **➞nervous system**. It is transversely oriented and the majority of its **➞neurites **are **axons **of **interneurons**.

**Discouraged terms: **none

**Figure 5:**
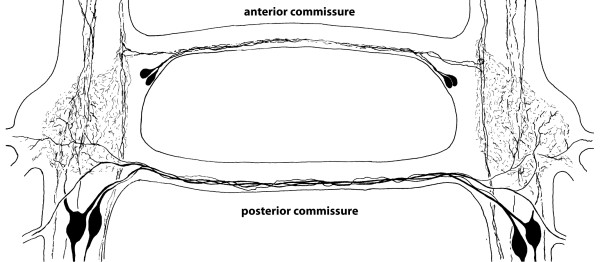
**Pair of ganglia in the rope-ladder-like nervous system of the branchiopod crustacean *Leptestheria dahalacensis***. Somata with neurites in the anterior and posterior commissures. Schematic drawing based on serotonin-like immunoreactivity. Modified from [198], with permission of Elsevier.

**Background/comment**: Commissures typically extend from left to right across the midline and connect longitudinal neurite bundles. In a **➞rope-ladder-like nervous system **they medially adjoin the **➞ganglia **of one **➞neuromere **across the midline (Figure 5). They may be embedded within the **➞neuropil **when the **hemiganglia **are close together (see also **➞tract**). In an **➞orthogon **they may take on the shape of a closed ring and are then called **ring commissures**.

### **{7} Compound eye**

A compound eye is an **➞eye**. It is part of a **➞nervous system **and consists of several to numerous almost identical components, the **➞ommatidia**. The sensory input of the compound eye is processed by at least two retinotopic **➞neuropils **connected to the **➞syncerebrum**.

**Discouraged terms: **facetted eye

**Background/comment: **Compound eyes are currently known to occur as lateral **cerebral eyes **in euarthropods such as Xiphosura within Chelicerata [76], Scutigeromorpha within Myriapoda [77], Branchiura [78], cirriped and ascothoracid larvae [79,80], Ostracoda Myodocopa [81], Branchiopoda [82,83] and Malacostraca (e.g., [84]) within Crustacea (summaries [85,86]), and most representatives of Hexapoda (e.g., [86]). Compound eyes share a single basal matrix, and **interommatidial pigment cells **are present between the ommatidia (see [79]). The **stemmata **in the larvae of holometabolous insects are modified compound eyes (e.g., [87,88]. The **lateral ocelli **in Pleurostigmophora are compound eyes as defined herein (for a detailed description see [89,90]). In certain Branchiopoda, the compound eyes are fused to form a single compound eye [83].

Eyes consisting of several units that have also been named compound eyes are also present on the tentacular crown in certain Annelida (many Sabellidae, some Serpulidae, see [91-95] and on the mantle edge in arcacean Bivalvia (Pterimorpha, Arcidae, see [93]). In arcean Bivalvia they act as alarm systems and are present in high numbers (in *Sabella*, for instance, up to 240 eyes are seen, each made up of 40-60 single unitscalled ocelii). The eyes (optic cushions) on the oral surface of Asteroida (Echinodermata), close to the base of the terminal tentacles, are also composed of a number of simple **ocelli **- as many as 80-200 in certain species [96,97]. We suggest avoiding the termini compound eyes and ommatidia when referring to non-arthropod eyes because the differences to those of arthropods overweigh the shared features.

### **{8} Connective**

A connective is a **➞neurite bundle**. It is part of a **➞nervous system**. It is completely or almost free of **somata **and interconnects **➞ganglia **longitudinally.

**Discouraged terms**: none

**Background/comment**: The majority of **➞neurites **in the connectives are **axons **of **interneurons **([18]; but compare **➞medullary cord**).

### **{9} Eye**

An eye is a **➞sensory organ**. It is part of a **➞nervous system **and consists of at least one **➞photoreceptor cell **and one separate **pigmented supportive cell**. An eye allows directional access of light to the photosensitive structures.

**Discouraged terms:** photoreceptor, ocellus

**Background/comment: **Not only does an eye allow light intensity to be measured, it also makes it possible to discriminate the direction of light. This is essential for **phototaxis**, the movement towards or away from a light source. In general, an eye consists of at least two and often numerous cells of two types: photoreceptor cells and pigmented supportive cells. The latter serve as shading structures and are crucial for the directional guidance of light to the photosensitive structures. Other cell types acting as light guiding structures may also be present. Some authors use the term eye only for those photoreceptive organs which are capable of producing an image. However, the evolution of photoreceptive organs is a story of a stunning increase in complexity, making it hard to find an objective border between "true" eyes and "proto-eye" precursors.

**Figure 6:**
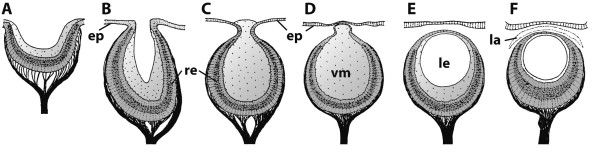
**Morphological sequence of different types of multicellular eyes exemplified by gastropod eyes**. **A**. Eye pit of *Patella *sp. **B**. Eye cup of *Pleurotomaria *sp. **C**. Pinhole eye of *Haliotis *sp. **D**. Closed eye of *Turbo creniferus*. **E**. Lens eye of *Murex brandaris*. **F**. Lens eye of *Nucella lapillus*. Abbreviations: ep = epidermis; la = lacuna; le = lens; re = retina; vm = vitreous mass. Modified from [101], with permission of Springer.

**Figure 7:**
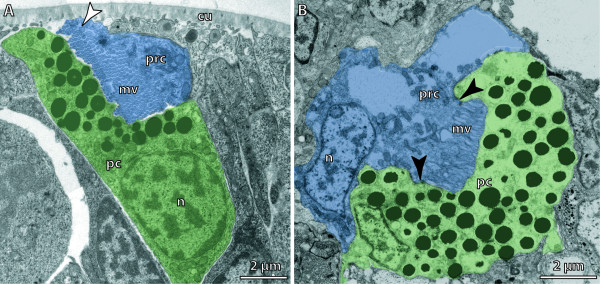
**Bicellular eyes (ocelli)**. Receptor cells are labelled blue and supportive cells are labelled green. **A. **Larval eye in a trochophore of *Platynereis dumerilii *(Annelida). Eye cavity communicates with exterior via a small pore (arrowhead). [TEM micrograph. Manually labelled.] **B. **Adult eye of *Protodrilus oculifer *(Annelida) composed of two cells. Arrowheads point to junctional complexes sealing the extracellular cavity formed by the photoreceptor cell and the pigment cell. [TEM micrograph. Manually labelled.] Abbreviations: cu = cuticle; mv = microvilli; n = nucleus; pc = pigment cell; prc = photoreceptor cell. Originals: G. Purschke.

Eyes of different kinds are found in almost every eumetazoan taxon (Figure 6; see [94,95,98-108]). The evolution of this diversity very likely started with only one multifunctional cell type, a condition that is observed in extant poriferan and cnidarian larvae, for example, which employ multifunctional cells with rhabdomeric photosensitive structures, shading pigment granules and locomotory cilia [109-112]. It is assumed that a multifunctional cell type diversified via functional segregation into sister cell types that were specialized in sub-functions such as **photoreception **on the one hand and shading of these photoreceptive structures on the other [111]. This led to the minimal eye (adhering to the definition given herein) being made up of only two cells: one photoreceptor cell and one supportive cell with shading pigment (Figure 7; [94,103,113]).

**Figure 8:**
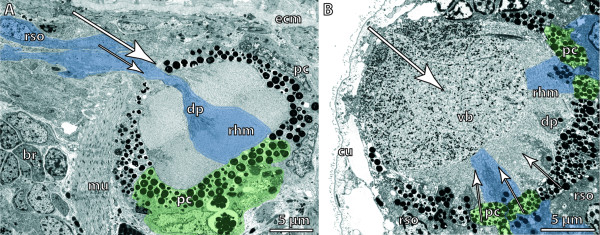
**Inverse and everse invertebrate eyes**. Large arrows indicate direction of incoming light, small arrows indicate orientation of light-sensitive processes of receptor cells. Some receptor cells are labelled blue and some supportive cells are labelled green. **A**. Pigment cup eye with inverse design of retina in a triclad flatworm, *Schmidtea mediterranea*. Dendritic processes of photoreceptor cells enter the eye cup through the opening of the pigment cup; the latter exclusively formed by pigmented supportive cells. Somata of photoreceptor cells lie in front of the opening of the eye cup. [TEM micrograph. Manually labelled.] **B**. Pigment cup eye with vitreous body or lens and everse design of retina in a polychaete, *Gyptis propinqua*, Phyllodocida. Dendritic processes of photoreceptor cells pass through the pigment cell layer. Note shading pigment within the dendritic processes. [TEM micrograph. Manually labelled.] Abbreviations: br = brain; cu = cuticle; dp = dendritic processes of photoreceptor cell; ecm = extracellular matrix; mu = muscle fibre; pc = pigment cell; rhm = rhabdomeric microvilli; rso = soma of receptor cell; vb = vitreous body. Originals: A: C. Kock; B: G. Purschke.

There are several types of structurally more complex eyes. These range from simple types called **pigment-cup eyes, ocelli **and **prototype eyes **[105], which comprise only **pigment cells **and photoreceptor cells, to highly sophisticated eyes that possess different kinds of light-guiding structures such as adjustable **lenses **and **irises**, as found in cephalopods and vertebrates, for example. However, highly developed lens eyes are not restricted to these "higher" taxa and may even occur in cnidarians, where lens eyes are part of the **rhopalia **in the medusae of Cubozoa [114]. A distinction is often made between the following morphological types of multicellular eyes (arranged in a hypothetical evolutionary transformation series): eye pit, eye cup, pinhole eye, closed eye, lens eye (Figure 6; [101]). In closed eyes and lens eyes a **cornea **may be developed. One specific eye type is the **➞compound eye **of arthropods. In multicellular eyes, photoreceptor cells usually form an epithelium, either exclusively or together with the pigmented supportive cells (depending on whether or not they carry shading pigment themselves). An epithelium comprising photoreceptor cells is called a **retina**. An **everse (converse) eye **is characterized by a retina in which the light-sensitive parts of the photoreceptor cells face the incoming light and are directed away from the concave surface of the eyecup (Figure 8B; [103]). In an **inverse eye **the light-sensitive parts of the photoreceptor cells face away from the incoming light or are directed towards the concave surface of the eyecup (Figure 8A; [103]). Due to functional constraints, a bicellular eye (Figure 7) is always an inverse eye, whereas multicellular eyes may be either of the two types, depending on the mode of development (Figure 8; [94,95]). An iris adjusts the opening of the eyecup according to the intensity of light and is usually composed of pigment and muscle cells. A **lens **permits the formation of an image on the retina of the eye. However, the distinction between lens and **vitreous body **("Füllmasse") is often not clear because functional investigations are often lacking (Figure 8B). Behind the photoreceptor cells, certain eyes may contain reflective cells characterized by membrane-bound crystalline platelets or reflective pigment granula which reflect light towards the photoreceptor cells to increase (the probability of) photon detection. Reflective cells are usually an adaptation to poor photic conditions. In larger eyes they are organized as an epithelium (**tapetum**). Such cells occur sparsely but are widely distributed among metazoans [115,116]. The substances most commonly reported to be the active component of reflective cells are guanine and pteridine.

Eyes situated in or close to the **➞brain **are commonly called **cerebral eyes **[103], though several examples of **extracerebral eyes **situated outside the brain and the condensed portion of the nervous system exist. Well-known examples are the eyes of the mantle edge in certain bivalves (Arcidae; see [93]), or polychaete tentacular (Sabellidae), segmental (Opheliidae, Syllidae) or pygidial eyes (Sabellidae) which occur in certain Annelida (see [95] for examples). Other examples are the optic cushions in Asteroida (Echinodermata).

An ocellus is nothing other than a diminutive eye. It is impossible to draw a clear distinction between an ocellus and an eye due to the impossibility of forming an unambiguous definition (see above; Figure 7A; [95]). In Arthropoda, the term ocellus is used for certain **➞median eyes **and for various **lateral eyes **which are considered to be modified **➞ommatidia **or **stemmata **(in particular in Myriapoda and Insecta; see [88]). The origin of the **lateral ocelli **in Arachnida remains an open question [88]. The eyes in Onychophora are also termed lateral ocelli [117,118]. Eye-like structures without shading pigments are frequently called **unpigmented ocelli**, although strictly speaking they are not eyes because they are not capable of detecting the direction of light, just the intensity. These structures are composed of photoreceptor cell(s) and supportive cell(s) without shading pigment granules.

Eyes occurring in planctonic larvae are called **larval eyes**. They are formed early in embryonic development and are found in the larvae of Hemichordata and Ascidiacea and in the larvae of lophotrochozoan taxa (Figure 7A). These eyes are composed of a limited number of cells (rarely more than 2-3) and are thus often called ocelli too. Their structure is comparatively well-known (for Mollusca see, for example, [119,120], for Platyhelminthes see, for example, [121] and for Polychaeta see, for example, [122,123]; molecular characterization is best studied in the polychaete *Platynereis dumerilii *[113,122,124]. During development, **adult eyes **are usually formed after the larval eyes are functional [95,105,120,122]. Apart from their simple structure, larval eyes are characterized by their molecular fingerprint and can thus be distinguished with certainty from adult eyes (Arendt et al., unpublished information). However, a structural distinction between a persisting larval eye and a newly developed miniaturized adult eye is not always discernible [125,126].

### **{10} Ganglion**

A ganglion is a cluster of **➞neurons**. It is part of a **➞nervous system**. It may include **➞glial cells**. The neurons are arranged in a specific constellation: neuronal **somata **are concentrated at the surface, thus forming a **cell cortex**, and **➞neurites **are concentrated in the centre of the ganglion to form the **➞neuropil**. A ganglion is a distinct unit but several ganglia may be anterio-posteriorly joined by **➞connectives **or transversally by **➞commissures**.

**Discouraged terms**: none

**Figure 9:**
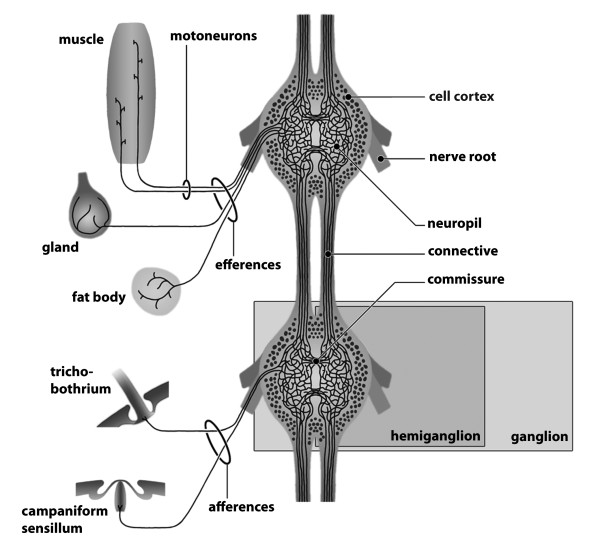
**Schematic presentation of two ganglia in the rope-ladder-like nervous system**. Original: C.M. Heuer.

**Background/comment**: The somata form a cell cortex that may be loosely or tightly packed and one or several cell layers thick but that is usually clearly demarcated (Figure 9). The cell cortex in Protostomia is dominated by **unipolar neurons**. Generally, there are no **➞synapses **in the cell cortex (but exceptions exist, e.g., in Arthropoda). The **primary neurite **of each neuron is directed inwards and, together with **dendrites **and a large number of **axons **of local **interneurons**, forms the neuropil of the ganglion, in which the synapses are located [18]. The neuropil may be loosely textured without defined regions or may be separated into neuropil partitions and include **➞tracts**. A ganglion may give rise to **➞nerves **which connect it to peripheral targets. In a **➞rope-ladder-like nervous system**, several ganglia may be antero-posteriorly joined by connectives (Figure 9). In the rope-ladder-like nervous system of many arthropods, bilaterally arranged pairs of ganglia transversely linked by commissures are present (Figure 5, 9). If bilaterally paired ganglia are fused at the midline, the partitions of this fused, single ganglion are called **hemiganglia**. It is important to stress that local swellings of a **➞medullary cord **do not qualify as ganglia as defined here. Neither does the so-called 'caudal ganglion' in Priapulida [127].

The protostome-centered definition offered above covers only a fraction of the anatomical structures that have been termed ganglia in (non-vertebrate) deuterostomes. These include the **cerebral ganglia **(or sensory ganglia) and the visceral ganglia in tunicate larvae [61,128] and the dorsal (or central or cerebral) ganglion in Pterobranchia [60,63] and adult Tunicata [61,62]. Contrary to our definition, the term 'ganglion' as currently used in deuterostomes is not restricted to a particular arrangement of neuropil and somata. Another difference is that in deuterostome ganglia, **multipolar neurons **are frequently present in addition to unipolar neurons, e.g., in the dorsal ganglion of salps [61,62]. We suggest using the term **➞brain **not only for the 'dorsal ganglia' of adult Tunicata and Pterobranchia (Figure 3D), but also for the larval 'cerebral ganglion' of tunicates. The 'visceral ganglion' of tunicate larvae is part of the neurulated nervous system (see **➞neural tube**), or more precisely, of the structure that is traditionally called the **central nervous system **in vertebrate morphology. As a result, in analogy to vertebrate morphology, the term 'visceral nucleus' or 'motor nucleus' is recommended.

Neuron concentrations in the nervous systems of Echinodermata, both larval [129,130] and adult [131], are also called ganglia. Because a central nervous system has not been identified in echinoderms nor a clear anatomical definition of a central nervous system provided for Protostomia, we should not apply the vertebrate-centred definition to Echinodermata. Our own definition applies to the repetitive ganglia present in the arms of ophiuroid brittle stars at least [132]. The apical concentrations of somata in the larval stages of Enteropneusta are called apical ganglia [133,134]. In line with the definition suggested for **➞apical organ **in the present work, we discourage the use of the term apical ganglion in these cases and suggest replacing it by apical organ.

In vertebrate anatomy a ganglion is any condensation of neuronal somata outside of the central nervous system and is to be distinguished from concentrations of neuronal somata within the central nervous system [135]. The latter are generally referred to as nuclei [136]. Though the term ganglion is, nevertheless, sometimes applied to concentrations of somata within the central nervous system, as in the case of the habenular ganglion or the basal ganglion [137,138], this use of the term is discouraged.

### **{11} Ganglion mother cell**

A ganglion mother cell is a **➞neuronal precursor**. It is part of a developing **➞nervous system. **It is generated by an asymmetrical division of a **➞neuroblast**. It divides once to produce **➞neurons **and/or **➞glial cells**.

**Discouraged terms: **none

**Background/comment: **So far, ganglion mother cells have only been described in hexapods [139,140] and malacostracan crustaceans [141,142].

### **{12} Glial cell**

A glial cell is a cell. It is part of the **➞nervous system. **A glial cell interacts closely with **➞neurons **by providing nutrients, removing the waste products of neuronal metabolism, electrically insulating the neurons and controlling the passage of substances from the blood to the neurons. It also supports, via its cytoskeleton, the structural arrangement of the cellular components of the **nervous tissue**.

**Discouraged terms:** neuroglia, supportive cell

**Figure 10:**
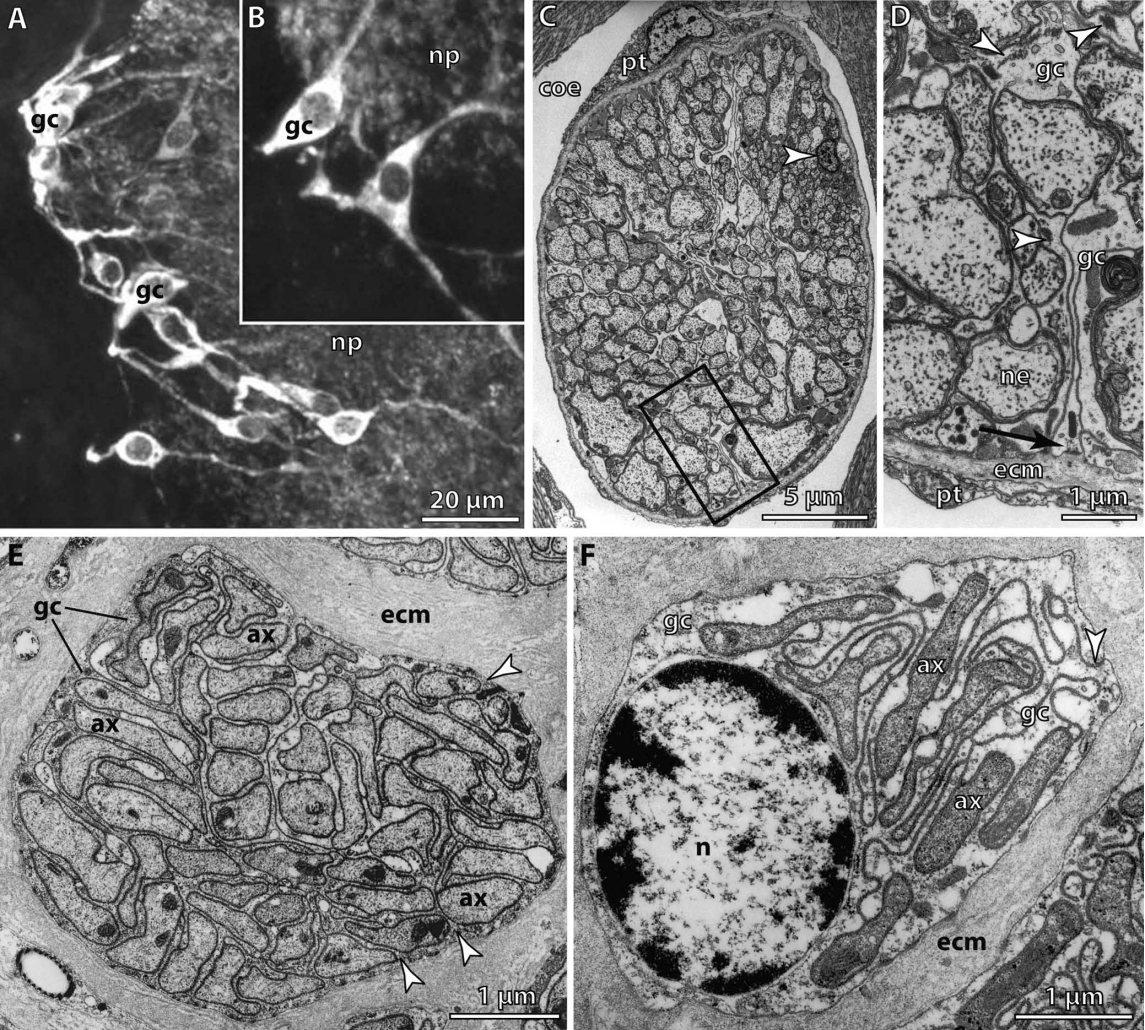
**Glial cells**. **A, B**. Ensheathing glial cells surrounding a neuropil in the brain of the terrestrial hermit crab *Coenobita clypeatus*. [Glutamine-like immunoreactivity.] **C, D**. Nuchal nerve in the opheliid polychaete *Armandia polyophthalma*. **C**. Entire nerve with groups of neurites separated by glial cell processes. Arrowhead points to soma of glial cell. [TEM micrograph.] **D**. Enlargement of boxed area from C. Glial cells attached to extracellular matrix (arrow). Arrowheads point to bundles of intermediate filaments. [TEM micrograph.] **E, F**. Optic nerve of *Scolopendra *sp. in cross section. **E**. Axon bundle with each axon separated from its neighbours by a glial cell process (arrowheads). [TEM micrograph.] **F**. Glial cell ensheathing axons with thin enrolled processes. Arrowhead points to junction of cell processes from both sides. [TEM micrograph.] Abbreviations: ax = axon; coe = coelom; ecm = extracellular matrix; gc = glial cell; n = nucleus; ne = neurite; np = neuropil; pt = peritoneum. A, B: From [314], creative common license of BMC; Originals: C, D: G. Purschke; E, F: C.H.G. Müller.

**Figure 11:**
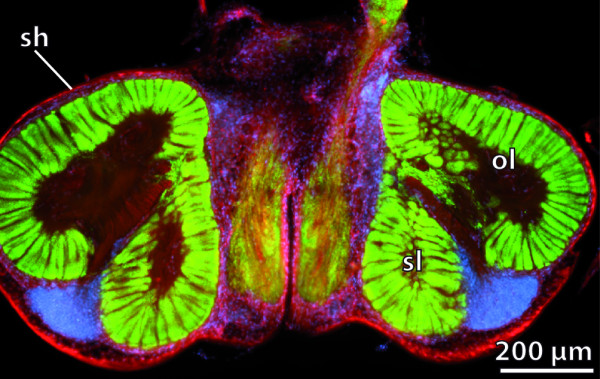
**Sheath**. Vibratome section of the brain of the terrestrial hermit crab *Coenobita clypeatus*. The sheath is shown in red, somata in blue, neuropil in green. [Synapsin-like immunoreactivity (green) combined with nuclear (blue) and actin stains (red).] Abbreviations: ol = olfactory lobe with olfactory glomeruli; sh = sheath; sl = side lobe. From [314], creative common license of BMC.

**Background/comment**: It is important to stress that glial cells are a heterogeneous class (Figure 10). Because of their role in metabolism glial cells usually contain stores of glycogen. The supportive glial cell is a type present in most invertebrates. It gives rise to processes and lamellae specialized in providing mechanical support. These processes often surround and ensheath single **➞neurites **or **➞neurite bundles **and - except in arthropods - contain intermediate filaments (Figure 10C, D). Within a neurite bundle, single **axons **may be separated from each other or form small units with a common glial **sheath **(Figure 10C, D, E). A sheath might also surround the **➞brain **and **➞ganglia **(Figure 11). The sheath is composed of an outer acellular layer, the **neurilemma**, and a layer of glial cells which underlies the fibrous material of the neurilemma and forms the **perilemma **(synonym **perineurium**) [143]. Neurite bundles that are not associated with glial cells are also common in various taxa of invertebrates. Furthermore, where there are intracerebral blood vessels, an additional role of glial cells is to provide a tight and relatively impermeable barrier (the blood-brain barrier) to prevent the diffusion of substances from the blood to the neurons [144].

### **{13} Globuli cell**

A globuli cell is a **➞neuron**. It is part of a cluster of other globuli cells. It possesses a minute amount of cytoplasm and a nucleus containing condensed chromatin. The **somata **of globuli cells are densely packed and easily discernable from other neighbouring neuronal somata due to their small diameter.

**Discouraged terms: **none

**Figure 12:**
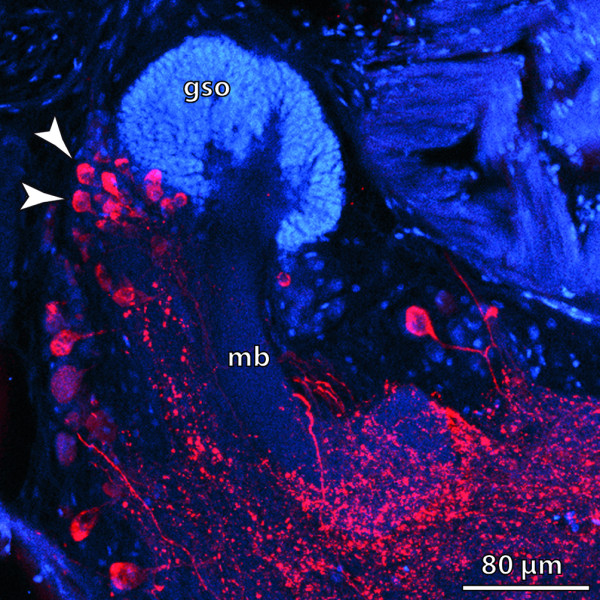
**Mushroom body in the polychaete *Nereis diversicolor***. Globuli cell somata form a dense aggregation and are pronouncedly smaller in diameter than neighbouring neuronal somata (arrowheads). The globuli cells are associated with the mushroom body. [Horizontal section. Double labelling showing FMRF-amide-like immunoreactivity in red and DAPI nuclear stain in blue.] Abbreviations: gso = globuli cell somata; mb = mushroom body. Original: C.M. Heuer.

**Background/comment: **Globuli cells have been described in the **➞brain **of Platyhelminthes [145], Nemertini [54], Mollusca [18,146], Polychaeta (Figure 12; [147]), Onychophora [42,67] and Euarthropoda [54]. One **➞neuropil **associated with globuli cell clusters is the **➞mushroom body **in Insecta [148] and Polychaeta [147]. In Insecta, the globuli cells which constitute the mushroom bodies are frequently referred to as **Kenyon cells**. The optic neuropils [149] and **hemiellipsoid bodies **in Decapoda [150] are also associated with globuli cell clusters.

### **{14} Lateral accessory lobe**

The lateral accessory lobe is a **➞neuropil**. It is part of the **➞central complex **(see Figure 4). A pair of lateral accessory lobes is located slightly posterior to the **➞central body**. Descending and ascending **➞neurons **of the lateral accessory lobes establish connections between the **➞central complex **and the postoral neuropils.

**Discouraged terms:** ventral body, lateral lobe

**Background/comment: **Anatomical and physiological evidence suggests that the lateral accessory lobes facilitate communication between the central complex and the motor centres in the thoracic **➞ganglia**. In addition, they also appear to connect other brain centres with the postoral neuropils [151]. Use of the abbreviatory term **lateral lobes **is discouraged to avoid confusion with an identical term that is used in a different context in molluscan neuroanatomy (Bivalvia: [152]; Gastropoda: [153]; Cephalopoda: [154]).

### **{15} Median eye**

A median eye is an **➞eye**. It is part of a **➞nervous system **and connected to a paired or unpaired median anterior **➞neuropil **of the **➞syncerebrum **by one or several **median eye nerve(s)**.

**Discouraged terms:** frontal ocellus, median ocellus

**Background/comment: **This term covers the various kinds of **➞nauplius eye**, the **frontal ocelli **in Hexapoda, the two **median ocelli **in Arachnida and Xiphosura (in the latter taxon, two additional median eyes might be present; [155]), and the four median ocelli present in Pycnogonida [156]. The term **ocellus**, however, is also used for various **lateral eyes **which are considered to be modified **➞ommatidia **or **stemmata **(in particular in Insecta; see [88]) and is discouraged herein. Median eyes are absent in Myriapoda.

**Figure 13:**
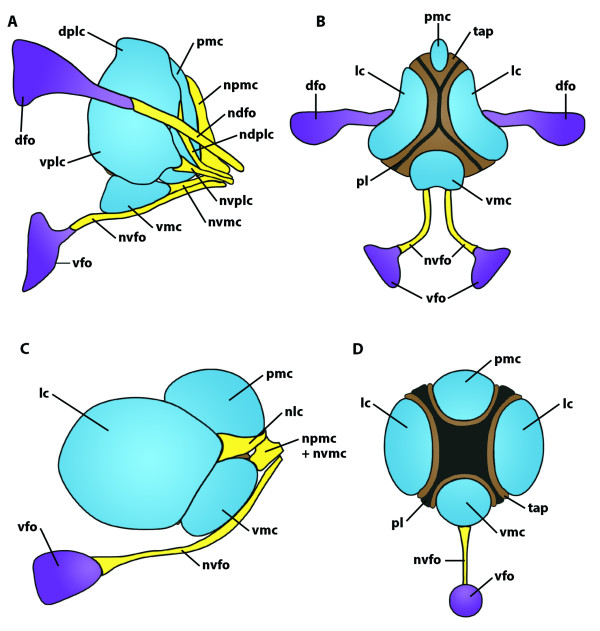
**Nauplius eye and frontal organs in two different branchiopod crustaceans**. **A, B:**. *Lynceus tatei *(Laevicaudata, Lynceidae). **A**. Lateral view (anterior is left). **B**. Frontal view (dorsal is up). **C, D: ***Cyclestheria hislopi *(Cyclestherida)**. C**. Lateral view (anterior is left). **D**. Frontal view (dorsal is up). Abbreviations: dfo = dorsal frontal organ; dplc = dorsal portion of the lateral cup; lc = lateral cup; ndfo = nerve connection between nauplius eye center and dorsal frontal organ; ndplc = nerve connection between nauplius eye center and dorsal portion of the lateral cup; nlc = nerve connection between nauplius eye center and lateral cup; npmc = nerve connection between nauplius eye center and posterior medial cup; nvfo = nerve connection between nauplius eye center and ventral frontal organ; nvmc = nerve connection between nauplius eye center and ventral medial cup; nvplc = nerve connection between nauplius eye center and ventral portion of the lateral cup; pl = pigment layer; pmc = posterior medial cup; tap = tapetum layer; vfo = ventral frontal organ; vmc = ventral medial cup; vplc = ventral portion of the lateral cup. Modified from [158], with permission of Elsevier.

In his seminal review, Paulus [86] suggested four median eyes for the ground pattern of Euarthropoda, though this has often been disputed. Mayer [118] suggested three median eyes to be part of the ground pattern of Tetraconata on the basis of the common presence of three median eyes in Hexapoda, e.g., in Archaeognatha, Zygentoma, and Pterygota (see [86]) and most crustaceans. Only representatives of the Phyllopoda possess a **four-partite (nauplius) eye **which, however, might represent the derived condition (Figure 13; [157,158]). On the basis of his argument that the '**lateral ocelli' **[117] in Onychophora are in fact homologous to median eyes, Mayer [118] suggested the presence of two median eyes to be part of the ground pattern of Arthopoda, a conclusion which is also supported by the presence of only one pair of median eye nerves in Xiphosura and Pycnogonida. In Xiphusura, the median eye nerves, which in the adult carry **afferents **from the median ocelli and the median rudimentary **photoreceptors**, terminate in the paired 'ocellar ganglia' (better: ocellar neuropils) in the anterior medial part of the **protocerebrum **[155]. A paired optic neuropil is also present in Pycnogonida [159]. In crustaceans, the nauplius eye centre is unpaired [160] but early anlagen appear paired [161].

### **{16} Medullary cord**

A medullary cord is a **➞nerve cord**. It is part of a **➞nervous system **and consists of a longitudinally extending central **➞neuropil **surrounded by a **cell cortex **consisting of neuronal **somata **distributed along its entire length. It may contain **➞glial cells **and **➞receptor cells**. A medullary cord is not divided into **➞ganglia **and soma-free **➞connectives**.

**Discouraged terms:** Markstrang

**Background/comment: **The presence of soma-free connectives (Figure 9) distinguishes a nervous system with ganglia from a nervous system with medullary cords [18]. The Onychophora are a typical example of Arthropoda with two medullary cords [162,163].

### **{17} Mushroom body**

A mushroom body is a **➞neuropil**. It is part of a **➞brain**. Mushroom bodies are paired and have a lobed shape. A mushroom body is composed of **dendrites **and parallelly arranged **axons **made up of thousands of **intrinsic neurons **(**➞neuron**) of the **➞globuli cell **type.

**Discouraged terms:** corpora pedunculata

**Background/comment: **Dujardin [164] was the first to describe mushroom bodies in Insecta, terming them corps pédonculés due to their resemblance to the fruiting bodies of fungi. Flögel [165] later defined criteria for identifying mushroom bodies across insect species; these criteria form the basis of the definition given above. The morphology of the cells which make up mushroom bodies (the 'globuli cells', or **Kenyon cells **in Insecta) was described in detail by Kenyon [166,167]. Kenyon subdivided the insect mushroom bodies into a **calyx **region - formed by dendritic branches of Kenyon cells, a **pedunculus **(peduncle) - formed by the parallel axons, and an arrangement of lobes. The first systematic surveys of the occurrence of mushroom bodies were conducted by Holmgren [40], and later by Hanström [54,168], who identified mushroom body-like neuropils in polychaetes (Figure 12), Insecta, Myriapoda, Onychophora and Chelicerata. In the latter two taxa, the neuropils of the two hemispheres are confluent across the midline of the brain [42,67]. A cluster of lobular neuropils in the brain of Cephalocarida (Crustacea) was also termed 'mushroom bodies' [169]. Although this cluster is laterally connected to a group of small-diameter globuli cell somata, its neuroarchitecture clearly differs from that in insects.

### **{18} Nauplius eye**

A nauplius eye is an **➞eye**. It is part of a **➞nervous system**. It consists of a cluster of three or four **➞median eyes **that form a single structural unit but are separated from one another by pigment layers.

**Discouraged terms: **three-partite eye, four-partite eye

**Background/comment: **This kind of eye (see Figure 13) is restricted to Crustacea. It is the only eye in nauplius larvae and persists in many taxa to the adult stage [170,171]. The exact anatomy differs between taxa [160]. In phyllopod branchiopods, the nauplius eye consists of four median eyes (also called eye cups) (Figure 13); in all other taxa, three eye cups are present. In addition to an absorbing pigment layer, a **tapetum **layer is present in Maxillopoda (e.g., [171-173]) and Phyllopoda [158], though it is formed by anatomically different components in these two taxa. One significant difference in the structure of the nauplius eye between taxa lies in the orientation of the **sensory cells**, which are directed towards the light (everse eyes) in Malacostraca but towards the pigment layer (**inverse eyes**) in other Crustacea [160]. A nauplius eye is completely absent in Mystacocarida, Cephalocarida and Remipedia. It is also absent in some Malacostraca. In addition to the nauplius eye, other photosensoric **frontal organs **might be present. Elofsson [160] argues that all photosensoric frontal organs should be called **frontal eyes **and regards the nauplius eye as nothing other than a complex of three or four frontal eyes which which evolved several times independently as three-partite or four-partite eyes. According to our definition, however, the frontal organs (apart from those forming the nauplius eye) are not eyes at all because they consist of sensory cells only without supportive **pigment cells **being present. In this, they differ fundamentally from nauplius eyes, even in cases where the nauplius eye cups become separated from each other during development (e.g., in cirripeds, [174]). In certain Decapoda, the pair of dorsal frontal organs forms a functional unit with the three-partite nauplius eye. It has been suggested that the term nauplius eye *sensu lato *could be extended to this unit, which is certainly an eye as we define it. The nauplius eye cups and, if present, additional frontal organs send their **axons **to a median brain centre in the anterior margin of the **protocerebrum**, the nauplius eye centre. Lacalli [175] described this in detail in a copepod as being rectangular in shape and subdivided into three cartridges, each receiving **➞nerves **from one of the three eye cups. Both the outer envelope and the internal subdivisions of the nauplius eye centre arise as flattened processes from a single pair of **➞glial cells**.

### **{19} Nerve**

A nerve is a **➞neurite bundle**. It is part of a **➞nervous system. **It connects a condensed nervous structure with a given region in the periphery, i.e., with **➞receptor cells **(mechanoreceptors, hygroreceptors, chemoreceptors, **photoreceptors**) or **effectors **(glands, fat body, muscles) or both.

**Discouraged terms: **none

**Figure 14:**
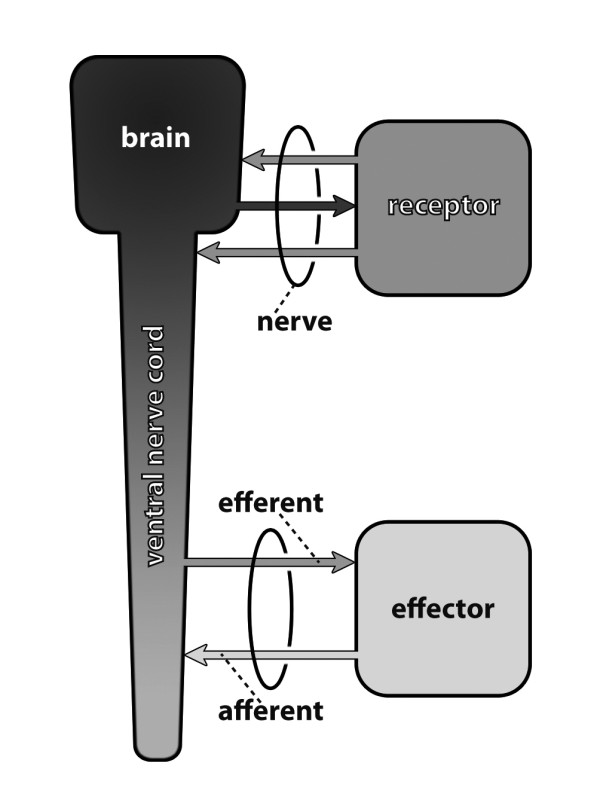
**Explanation of basic nervous system terminology**. Receptors are receptor cells or sensory organs, e.g., eyes, olfactory sensilla, or mechanosensilla. Effectors are, e.g., muscles, glands, or the fat body. Original: S. Harzsch, C.M. Heuer.

**Background/comment: **The term nerve can only be applied to metazoans, in which a condensed nervous structure (e.g., **➞ganglion**, **➞brain**) can be distinguished from more peripheral elements (in accordance with [18]; Figure 9). Typically, nerves are free of cell **somata **and are composed of **axons**: either the axons from receptor cells that are extended towards the centre (**afferents**) or the axons of **motoneurons **that target the periphery (**efferents; **[18]; Figure 14). In the arthropod literature, a nerve entering the **central nervous system **is sometimes called a **➞tract**. The term nerve as defined here is much more restricted than it is generally used in invertebrate neuroanatomical description and needs in particular to be distinguished from the more general term neurite bundle.

### **{20} Nerve cord**

A nerve cord is a cluster of **➞neurons**. It is the most prominent longitudinally extending condensed part of a **➞nervous system**.

**Discouraged terms: **none

**Background/comment**: In animals with an anteroposterior axis, a single prominent longitudinal **➞neurite bundle**, or a pair thereof, is often positioned dorsally or ventrally and extends longitudinally throughout the body. Such bundles are traditionally termed nerve cords and are important factors in concepts dividing Bilateria into animals with a **ventral nerve cord **(gastroneuralians) and those with a dorsal nerve cord (notoneuralians). Our definition of the nerve cord also works in relation to other longitudinal neurite bundles: only the most prominent of these bundles is called the nerve cord. A ventral nerve cord can be either paired or unpaired, and can be a **➞medullary cord **or contain **➞ganglia**.

### **{21} Nervous system**

A nervous system is a cluster of **➞neurons**. It comprises all neurons of an organism and may include additional **➞glial cells**. It may also include accessory cells, which, for example, serve as supportive structures, stimulus guiding structures, or protective structures.

**Discouraged terms: **none

**Background/comment**: The defining character of a nervous system is the presence of cells recognizable as neurons. A related term is **nervous tissue**. Several specialized macromolecules such as **receptors **or ion pumps or components such as vesicle molecules or enzymes involved in transmitter metabolism that are present in neurons have been detected in sponges but a morphologically discernable nervous system is not present (e.g., [176]; compare with **➞neuron**). In many cases, a distinction is made between the **central nervous system (CNS) **and the **peripheral nervous system (PNS)**. The CNS, according to Bullock and Horridge [18], is "... that part of the nervous system which forms a distinct principal concentration of cords or **➞ganglia **...." According to this definition, the grade of condensation of **➞neurites **into **➞neurite bundles **is the distinguishing feature of a CNS. Additionally, the term CNS implies a reference to the proximo-distal axis that defines the centre and periphery of an organism. Both definitions are problematic because strong neurite bundles can either occur intraepithelially (and therefore in the periphery of the organism) or subepithelially. Moreover, less dense neurite bundles may occur in the central part of an organism. The range of the degree of condensation, i.e., the diameter of a neurite bundle, is continuous, which sometimes makes it impossible to decide whether a neurite bundle should be considered CNS or PNS. In chordates, the term central nervous system is commonly used for the **➞neural tube **(Figure 3B). The distinction between a CNS and a PNS is usually thought to be characteristic of bilaterian animals, but the detection of condensed parts in the nervous system of cnidarians, especially medusae, poses additional problems when the grade of condensation is the only aspect taken into account. In this sense it is logical that such structures in medusae should be termed/allocated to the CNS (e.g., [43]). We suggest avoiding the terms CNS and PNS and characterizing a neurite bundle by (a) its size and (b) its location within the organism.

### **{22} Neural tube**

A neural tube is a **➞nerve cord**. It is part of a **➞nervous system**. It has a tubular structure and contains a central fluid-filled canal, the **neural canal**.

**Discouraged terms:** nerve tube

**Figure 15:**
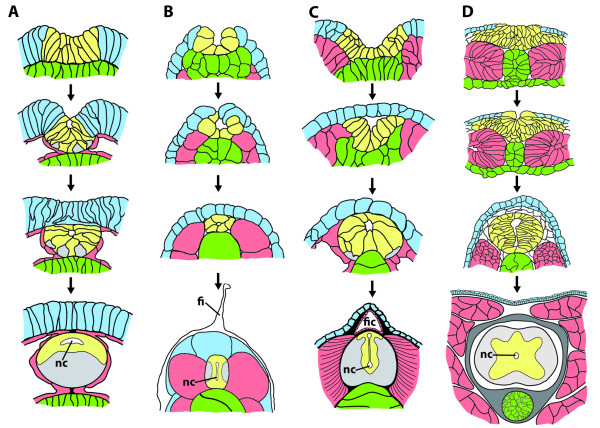
**Semischematic representations of the neurulation processes in different deuterostome taxa**. **A**. Neurulation in enteropneusts (*Saccoglossus kowalevskii*). **B**. Neurulation in ascidians (*Boltenia villosa, Molgula occidentalis*). **C**. Neurulation in cephalochordates (*Branchiostoma belcheri *and *B. lanceolatum*). **D**. Neurulation in blue amphibians (*Xenopus laevis*). Light blue = epidermis; green = endoderm and endodermally derived notochord; red = mesoderm; yellow = nervous tissue. Abbreviations: fi = fin; fic = fin chamber; nc = neural canal. A: Modified from [324]; B: Modified from [325,326], C: Modified from [324,327]; D: Modified from [328].

**Background/comment: **During development, the neural tube originates via a morphogenetic process in which a portion of the aboral epithelium becomes internalized (e.g., [137]). This process is called **neurulation**. The internalized ectodermal tissue differentiates into **nervous tissue **that forms the neural tube. The details of the internalization process may differ [177-179], with possible scenarios ranging from (i) the invagination of a longitudinal area of epithelium (Figure 15A, D: Tunicata, Amphibia), (ii) a sinking in of a neural plate that is overgrown by lateral extensions of the epidermis (Figure 15B, C: Cephalochordata, Enteropneusta), to (iii) the ingrowth of a compact longitudinal strand of the dorsal epidermis underneath the extracellular material (Teleostei). The result is always a neural tube that lies beneath the epidermis and is therefore surrounded by an extracellular matrix (Figure 3B). The neural tube contains a fluid-filled hollow central canal termed the neural canal which is lined by ciliated cells. In a throwback to its ontogenetic origin, the neural canal connects to the outside at the anterior end through the '**neuropore'**. In Chordata, the neural canal contains a mucous strand, 'Reissner's fibre', which originates from distinct anterior infundibular cells. Furthermore, in addition to the anterior neuropore, the posterior end of the neural canal connects via the '**neurenteric canal' **(Canalis neurentericus) to the intestinal tract [180]. The centralized part of the chordate nervous system can often be divided into two parts. The anterior part, the **➞brain**, is characterized by its larger transversal and dorsoventral diameter and/or a dilation of the central neural canal [138,181,182]. It is thus distinguished from the narrower and more uniform posterior part, the spinal cord (Craniota) or **neural cord **(Tunicata, Cephalochordata). In Ascidiacea, the subepidermal brain (often called the dorsal ganglion, see **➞ganglion**) is completely surrounded by an extracellular matrix and is derived in part from the anterior part of the larval neural tube. The more posterior part of the larval neural tube, including the visceral nucleus (see **➞ganglion**) is reported to become phagocytized [183]. In Thaliacea, the brain (often called dorsal ganglion, see **➞ganglion**) is also solid in adults but undergoes a stage where a neural tube with a hollow fluid-filled cavity and cilia is present [62].

### **{23} Neurite**

A neurite is a cell process. It is part of a **➞neuron**. Neurites are divided into **primary neurites**, **axons**, and **dendrites**.

**Discouraged terms:** nerve fiber, Nervenfaser, axis cylinder, nerve

**Background/comment: **Traditionally, the term 'neurite' has been used for "the main or longest process of a nerve cell" [18] or, mostly in vertebrates, as a synonym to 'axon' [18,184]. In insects, the term 'neurite' is often used to denote the single main process of **unipolar neurons **that connects the **soma **and the integrative part consisting of dendrites and axons [185]. Because of the ambiguity of the term and because in many invertebrates it is difficult to distinguish axons and dendrites on the basis of histological criteria and in the absence of electrophysiological data, Bullock and Horridge [18] rightly suggested that the term needs to be rejuvenated. We therefore suggest that all cell processes of neurons collectively be referred to 'neurites', a practice that has already been adopted in some studies of invertebrate **➞nervous systems **(e.g., [186,187]). The single main process emerging from the soma of unipolar neurons and connecting them to dendrites and axons is then called 'primary neurite'. Dendrites are those neurites of a neuron that receive stimuli/input. They may house postsynaptic components to allow them to receive axonal input from other neurons. Axons are those neurites of a neuron which house presynaptic components and which target the dendrites of other neurons or peripheral organs such as muscles, glands or fat bodies. We are convinced that this rejuvenated, clear terminology will encourage uniformity in the description of invertebrate nervous systems. It will also help solve conflicts such as those surrounding the **➞plexus **of cnidarian nervous systems, the same elements of which have been referred to as "nerve fibres" [18,186], "processes" [188] and "neurites" [186,189,190].

### **{24} Neurite bundle**

A neurite bundle is a cluster of **➞neurites**. It is part of the **➞nervous system**. The neurites are arranged in parallel to form a bundle.

**Discouraged terms**: none

**Background/comment: **Neurites can occur as single neurites or in neurite bundles. Neurite bundles are composed of a variable number of neurites. Traditionally, very thick neurite bundles are often termed the **➞nerve cord**.

### **{25} Neuroactive substance**

A neuroactive substance is a molecule. It is part of the **➞nervous system**. It is diffusible and influences the physiological state of **➞neurons **by interacting with a competent **receptor**.

**Discouraged terms**: none

**Background/comment: **Neuroactive substances either influence the electrophysiological state of a neuron directly via synaptic interactions (neurotransmission) or modify the response of neurons to external stimulation (neuromodulation). Neuroactive substances are classified according to their molecular structure [191,192]:

i) amino acids and their derivatives, which are known as **biogenic amines **(e.g., serotonin, histamine)

ii) **neuropeptides **(e.g., FMRFamide, allatostatin, tachykinin)

iii) gaseous molecules (e.g., nitric oxide, carbon monoxide)

**Figure 16:**
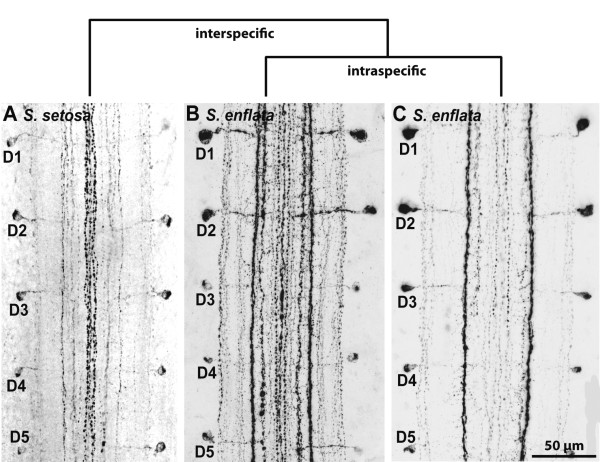
**Individually identifiable neurons in the ventral nerve center of the chaetognaths *Sagitta setosa *and *S. enflata***. D1-D5 label individually identifiable neurons. Note also the intraspecific and interspecific differences [FMRF-amide-like immunoreactivity.] From [329], with permission of Springer.

A large number of putative neuroactive substances have been identified in the **➞nervous system **of invertebrates (Annelida: [193]; Insecta: [191]; Cnidaria: [194]; Nematoda: [195]; Mollusca: [196,197]. In anatomical studies, neuroactive substances are usually identified on the basis of immunocytochemical investigations (e.g, see Figure 3 and Figure 16), without support from physiological and pharmacological studies. Immunocytochemistry cannot be taken as proof of the presence and physiological effect of a neuroactive substance, however. This is especially true if antibodies target epitopes such as RFamides which are shared by various members of a family of neuroactive molecules. Accordingly, neurons showing an immunopositive response to anti-FMRFamide should not be termed 'FMRFamidergic' but 'FMRFamide-like immunoreactive'. The localization of neuroactive substances that are present over a wide taxonomic range has frequently been the subject of comparative neuroanatomical studies (tachykinin: [64]; serotonin: [198]; FMRFamide: [199]; histamine: [200]).

### **{26} Neuroblast**

A neuroblast is a **➞neuronal precursor**. It is part of a developing **➞nervous system. **It is comparably large and acts as a stem cell. It divides asymmetrically and preferentially in one direction only, giving rise to smaller cells, the **➞ganglion mother cells**.

**Discouraged terms: **none

**Figure 17:**
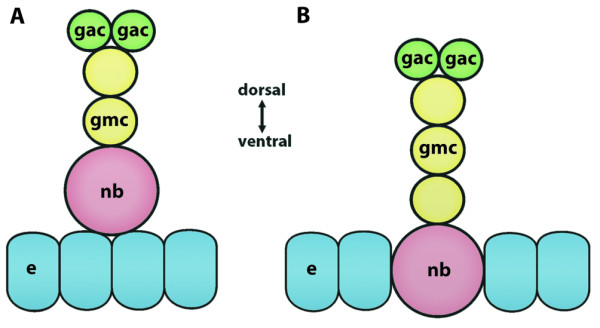
**Schematic representation of segmental neuroblasts and their progeny in insects and malacostracan crustaceans in cross section**. **A**. In insects, the neuroblasts detach from the ventral embryonic ectodermal layer and migrate into the interior of the embryo in dorsal direction. After this process they produce the ganglion mother cells which in turn divide to form ganglion cells (i.e., neurons or glia). **B**. In malacostracan crustaceans, the neuroblasts remain in the ectoderm, but the production of ganglion mother cells and ganglion cells shows the same pattern as in insects. Abbreviations: e = ectoderm; gac = ganglion cells; gmc = ganglion mother cells; nb = neuroblast. Modified from [142], with permission of the Royal Society in London.

**Background/comment: **The term neuroblast is often applied to neuronal precursors in general. Here, a strict definition restricted to large specialized stem cells is preferred. To date, neuroblasts have been found in representatives of Insecta and Malacostraca (Figure 17; e.g., [139-141,201-208]). In malacostracan crustaceans and insects it has been possible to identify and homologize individual neuroblasts with regard to their origin, gene expression and the lineage which give rise to **➞pioneer neurons **[139,140,142,209]. The situation in non-malacostracan crustaceans is somewhat ambiguous. Preliminary descriptions of the possible occurrence of neuroblasts still await confirmation [210-212].

The neuroblasts of Insecta differentiate after immigration from the ventral ectoderm (Figure 17A; [204]) whereas those of Malacostraca remain in the embryonic surface cell layer (Figure 17B; [141,213,214]. In addition to the neuroblasts involved in the formation of the ventral **➞ganglia **of the trunk, a corresponding cell type has been detected in the **➞brain **area of insects and malacostracan crustaceans [215,216]. However, in contrast to the neuroblasts of the forming trunk ganglia, brain neuroblasts do not bud their progeny into the inner part of the embryo but tangentially to the surface. In malacostracans, some neuroblasts have been described as dividing equally after producing several ganglion mother cells by unequal cleavage [217]. In other words, during equal divisions neuroblasts do not act as neuroblasts in a proper sense, though neuroblastic activity is continued afterwards. Nothing comparable has yet been observed in insects.

### **{27} Neuromere**

A neuromere is a cluster of **➞cells**. It is part of a developing **➞nervous system. **It consists of all the developing **nervous tissue **that is part of one of the several anterior-posterior repetitive units of the nervous system.

**Discouraged terms: **none

**Figure 18:**
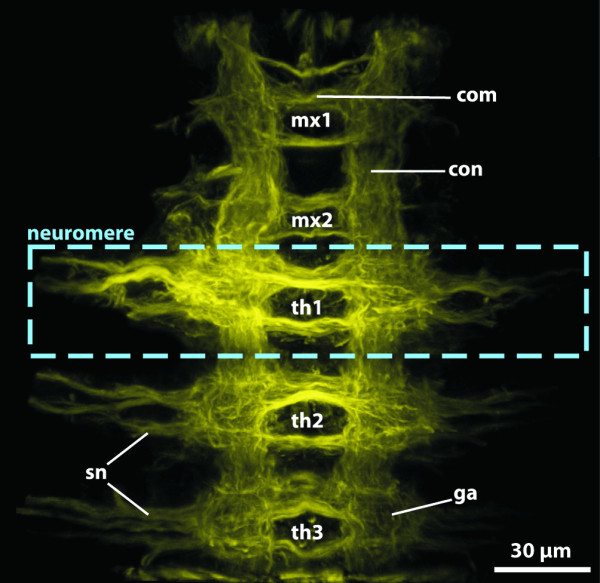
**Developing ventral nerve cord in *Triops cancriformes *(Crustacea, Branchiopoda)**. A neuromere consists of all developing nervous tissue that is part of one anterior-posterior repetitive unit of the nervous system, e.g., as marked here, thorax segment 1. Larval stage 3 in ventral view. [Acetylated α-tubulin immunoreactivity.] Abbreviations: com = commissure; con = connective; ga = ganglion; mx1-2 and th1-3 = position of segments of maxilla 1 and 2, and thoracopods 1 to 3, respectively. Original: M. Fritsch.

**Background/comment: **This term has its origin in developmental biology (e.g., [218]) and is herein restricted to embryos and larvae. In many arthropods, the **soma**-free **➞connectives **between the **➞ganglia **develop later on, whereas the embryonic segmental units of the nervous system - the neuromeres (Figure 18) - adjoin each other. In Arthropoda, molecular geneticists prefer to define body segments e.g., on the basis of the expression of the segment polarity gene *engrailed *in transverse stripes of the posterior portion of forming segments [207,219]. If no *engrailed *data are available, however, body segments are generally identified by morphologists on the basis of their external morphology, i.e., the anlagen of the limb rows or the trunk segments. The term neuromere refers to segments identified in both ways.

### **{28} Neuron**

A neuron is a cell. It is part of the **➞nervous system **and consists of a **soma **that gives rise to **➞neurites**, which conduct electric excitation in a directed way. A neuron communicates with other cells via **➞synapses**. Most neurons synthesize and secrete **➞neuroactive substances**.

**Discouraged terms:** nerve cell

**Figure 19:**
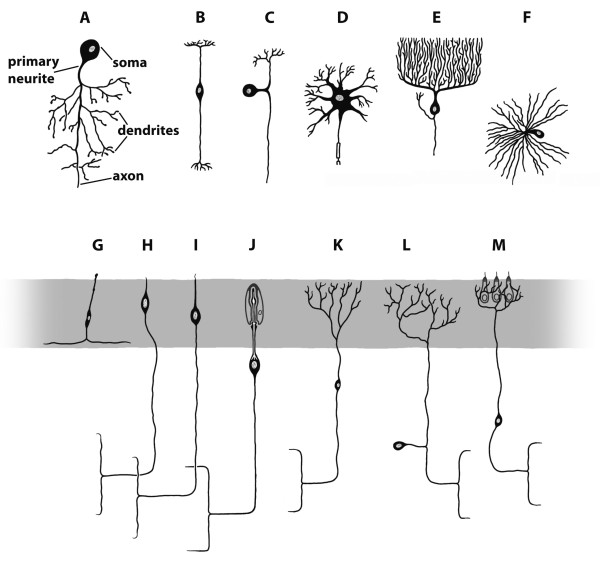
**Schematic representation of different types of neurons (modified from various sources)**. **A. **Unipolar neuron and terminology of different cell parts. **B. **Bipolar neuron. **C. **Pseudounipolar neuron. **D, E, F. **Multipolar neurons of different morphology. **G**. Bipolar receptor cell sending its axonal processes into an intraepidermal plexus. **H, I**. Bipolar receptor cells with a short distal (dendritic) process and with a soma embedded in the epithelium. Most common type in invertebrates. **J**. Bipolar receptor cell with elaborated distal process (arthropod scolopale). **K, L**. So-called free nerve endings with bipolar (K) and unipolar receptor cells as in vertebrates (L). **M. **Receptor cell showing a bipolar form connected by its dendritic processes with a group of epithelial cells specialized as receptor elements (so-called secondary sensory cells). Modified from [18], with permission of Freeman.

**Figure 20:**
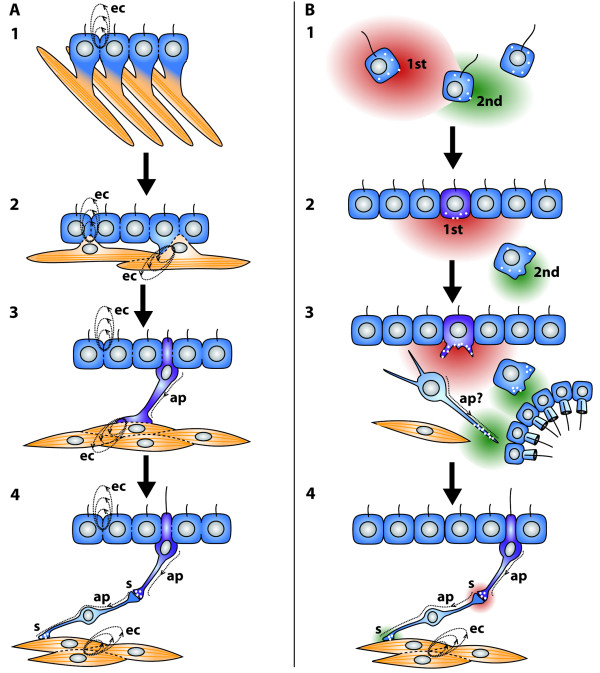
**Schematic comparison of two evolutionary scenarios for the nervous system (from **[223]). **A**. Neuro-muscular hypothesis [330]. (1) Primordial myoepithelium with electrically coupled cells. (2) Protomyocytes start to forsake the epithelium, sinking into the interior. (3) Protoneurons evolve, conveying excitation from the exterior to the myocytes. (4) Neurosensory cells and neurons evolve, which make use of action potentials. They are connected to one another and to the myocytes by chemically transmitting, polarized junctions. Electrical coupling persists in many epithelia and muscles. **B**. Paracrine-to-electrochemical-dominance transition hypothesis (modified from [331,332]) (1) Paracrine signaling in unicellular eukaryotes with signals of the first or second order. (2) Hypothetical intracorporeal paracrine signaling in early metazoans with cascaded paracrine signals: first-order signals originate from externally stimulated epithelial cells; these signals stimulate mesenchymal cells, which release second-order paracrine signals that might be the same substance (positive feedback) or another messenger (integration). (3) New cell types evolve, with the trophic effects of paracrine messengers leading to prolonged multipolar cells. Eventually, action potentials are present and secretion of messengers is compartmentalized within peripheral parts of the cells. (4) Polarized and compartmentalized cells evolve into neurosensory cells and neurons, with further concentration of messenger secretion into peripheral synapse structures and AP traveling over long distances (paracrine-to-electrochemical-dominance transition). Abbreviations: ap = action potential; ec = electrical coupling; 1st = primary chemical signal; 2nd = secondary chemical signal; s = synapse. A, B reprinted from [223], with permission of Wiley.

**Background/comment**: It is hard to find exclusive morphological or physiological criteria to define a neuron because features such as excitability, cell processes and the secretion of substances are also shared by other cell types such as gland cells and muscle cells. What defines a neuron is a combination of these features [18,220]. One important function of neurons is the directed conduction of excitation (reviews [221-223]). Historic aspects of the physiological neuron concept have recently been reviewed by Barbara [221]. The neuronal cell body is called the soma (synonym **perikaryon**). Neurons that only give off one neurite are called **unipolar neurons **(Figure 19A). This **primary neurite **connects the soma to the **dendrites **and **axons**. **Bipolar neurons **separately give rise to one axon and one primary dendrite (Figure 19B). In a **pseudounipolar neuron**, the primary neurite splits into an axon and a dendrite shortly after it exits the soma (Figure 19C). In **multipolar neurons**, one axon and/or many dendrites branch directly off the soma (Figure 19D, E, F). Neurons that target other neurons are called **interneurons**. **Intrinsic neurons **are interneurons whose neurites are confined to specific **➞neuropils**. **Extrinsic neurons **are interneurons that connect different neuropils. Neurons that target muscles are called **motoneurons**. Neurons are present in Ctenophora and Cnidaria (although it is currently disputed whether or not neurons may have evolved independently in these two taxa) and all other Eumetazoa [220]. Some sponge cell types share some of the molecular and physiological characteristics of neurons but do not entirely fulfil the criteria for neurons as defined here (discussed in [223-226]). The evolutionary emergence of neurons is a hotly debated topic (recent reviews e.g., [220,223,227]), and Figure 20 features two current hypotheses on this issue.

Neurons that can be individually recognized from animal to animal in one species or even in the animals of different species are called **individually identifiable neurons **(Figure 5, 16; [228]). These may be serially arranged (i.e., iterated along the anterior-posterior axis) and resemble iterated "clones" (Figure 16). Many of the recent studies on this topic rely on the foundations laid by Kutsch and Breidbach [229]. These authors presented a catalogue of features that can be used to examine the cellular characteristics of individually identifiable neurons in order to explore whether they are homologous between different arthropod taxa. The authors distinguish between interspecific homology (comparison of neurons between the animals of different species) and serial homology (repetitive, equivalent neurons in the different segmental **➞ganglia **of the animals of one species; Figure 16). Within Protostomia, individually identifiable neurons have been shown to be present in the **➞nervous system **of Arthropoda [185,228,230], Annelida [52,56,231-234], Nemathelminthes/Cycloneuralia [235,236], Mollusca [237,238], Platyhelminthes [239-241] and Gnathifera [242]. The presence of at least some individually identifiable neurons in Deuterostomia such as Tunicata [243-247] and Cephalochordata [248] indicates that the potential to establish individual identities may not only be present in the ground pattern of Protostomia. Serially arranged individually identifiable neurons are not only found in typically segmented Protostomia such as Annelida (including Echiurida) and Arthropoda, but also in unsegmented organisms such as representatives of Nematoda [236], Platyhelminthes [239-241], Chaetognatha [144], Sipuncula [249,250] and Priapulida [127] and in basal Mollusca [237,238].

### **{29} Neuronal precursor**

A neuronal precursor is a cell. It is part of a developing **➞nervous system. **It produces either further neuronal precursors or **➞neurons **or **➞glial cells**.

**Discouraged terms:** neuronal progenitor

**Background/comment: **Most neuronal precursors cannot be identified on the basis of morphological characteristics. The notable exception is the **➞neuroblast**, which is relatively large and divides asymmetrically (Figure 17). The term neuronal precursor as defined here excludes cells that directly transform into neurons or glia cells without further mitosis. This, for instance, is the case for the immigrating cells of various chelicerate embryos, which directly assume a neuronal appearance once they become detached from the embryonic ectoderm [251]. According to their position in the embryo, neuronal precursors can be designated more specifically, e.g., 'median precursor' in the *Drosophila *embryonic midline [252].

### **{30} Neuropil**

A neuropil is a cluster of **➞neurites**. It is part of a **➞nervous system **and forms a network of **dendrites **and **axons **where **➞synapses **are present and in which neuronal **somata **do not occur.

**Discouraged terms: **none

**Figure 21:**
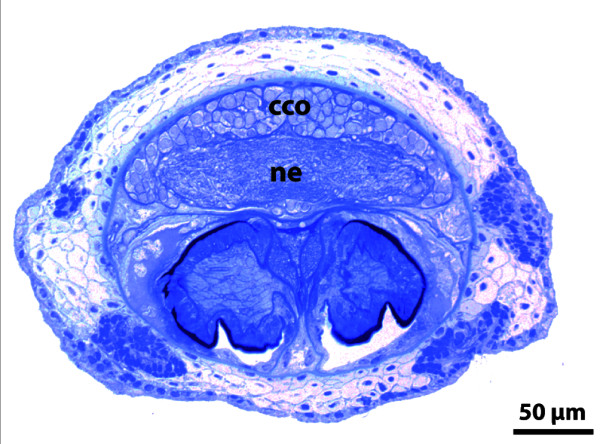
**Cross section through the head of the chaetognath *Ferrosagitta hispida***. The somata of the brain are arranged in a cell cortex surrounding the neuropil. Abbreviations: cco = cell cortex; ne = neuropil. Original: A. Sombke, G.L. Shinn, C.H.G. Müller, S. Harzsch.

**Figure 22:**
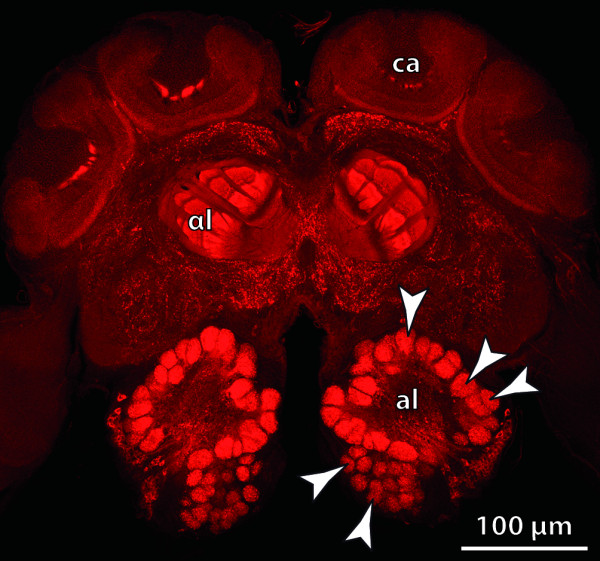
**Olfactory glomeruli (arrowheads) in the brain of the ant *Camponotus ocreatus***. The olfactory glomeruli are located within the antennal lobe. [Frontal section. Allatostatin-like immunoreactivity.] Abbreviations: al = antennal lobe; αl = alpha lobe of the mushroom body; ca = calyx. Original: R. Loesel.

**Background/comment**: Because of the synaptic interactions which take place in it, a neuropil is the principal region of integrative processing events [18]. The neuronal somata of the **interneurons **that extend their neurites into the neuropil are located outside the neuropil and may surround it in a **cell cortex **(Figure 21). However, **➞glial cell **somata, **➞tracts**, blood vessels and tracheae may be embedded within a neuropil. A neuropil can be further compartmentalized, e.g., by glial boundaries. The resulting partitions are also termed neuropils and may have been given specific names such as **➞central body **(Figure 4) or **➞olfactory glomeruli **(see compartments within **olfactory lobe **in Figure 11, **antennal lobe **in Figure 22).

### **{31} Olfactory glomerulus**

An olfactory glomerulus is a **➞neuropil**. It is part of a **➞nervous system**. An olfactory glomerulus is a clearly demarcated, dense neuropil in which olfactory receptor **➞neurons **terminate and form the first **➞synapses **of the olfactory pathway.

**Discouraged terms**: none

**Background/comment: **Olfactory glomeruli occur in many metazoan taxa. They provide a means for the spatial representation of chemosensory information (reviewed by [253]). Olfactory glomeruli are usually arranged in clusters (Figure 11, 22). Despite architectural similarities between different taxa, olfactory glomeruli are not necessarily located in comparable positions in the **➞nervous system**. The olfactory glomeruli of Tetraconata are located in the **deutocerebrum**, for example, while in Onychophora they are situated in the **protocerebrum **and in Chelicerata they occur in the **➞ganglion **of whichever segment bears an appendage equipped with odour receptors [42]. Olfactory glomeruli have been described in representatives of major metazoan lineages: Annelida [254], Arthropoda [255], Mollusca [256], and Craniota [257].

### **{32} Ommatidium**

An ommatidium is an **➞eye**. It is the smallest morphological and functional unit of the **➞compound eye **and consists of a usually limited and often constant number of rhabdomeric **➞photoreceptor cells**, **cornea-secreting epithelial cells**, and **interommatidial pigment cells**, and may additionally contain **crystalline cone **cells.

**Discouraged terms: **none

**Figure 23:**
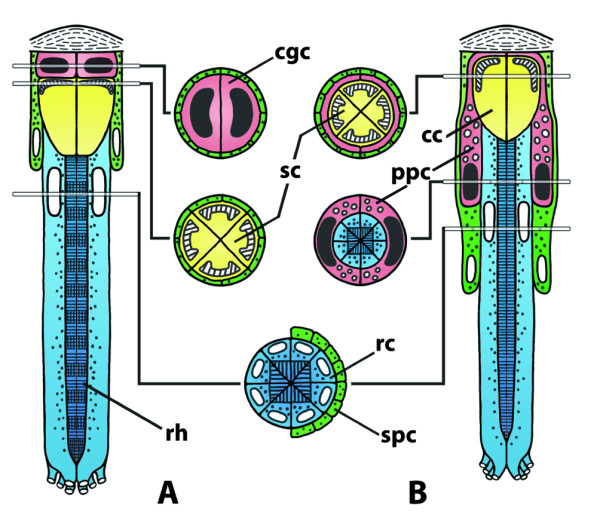
**Ommatidia in Crustacea (A) and Hexapoda (B)**. Cross sections are indicated by arrows. Both ommatidium types are identical in cell types and cell numbers: two corneageneous cells in Crustacea and two primary pigment cells in Hexapoda, four Semper cells forming a crystalline cone, eight retinula cells forming a closed rhabdom. Abbreviations: cc = crystalline cone; cgc = corneageneous cells; ppc = primary pigment cells; rc = retinula cells; rh = rhabdom; sc = Semper cells; spc = secondary pigment cells. Modified from [87], with permission of Wiley.

**Figure 24:**
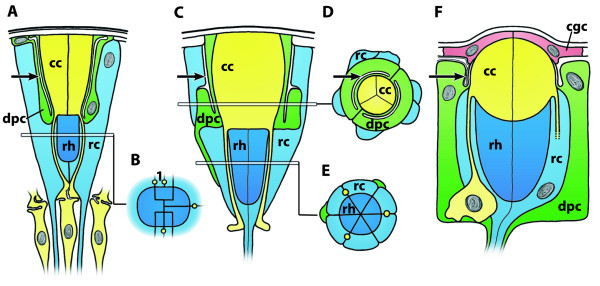
**Ommatidia in three different Maxillopoda (Crustacea)**. **A, B. ***Argulus foliaceus *(Branchiura). **A. **Overview. **B. **Transverse section through the rhabdom. Retinula cell between two cone cell processes. Eighth retinula cell not shown. **C, D, E. ***Balanus crenatus *(Cirripedia). **C. **Overview. **D. **Transverse section through the crystal cone and the distal pigment cells. **E. **Transverse section through the rhabdom. Three cone cell processes are present. **F. ***Cypridina norvegica *(Ostracoda). Note the extracellular space (arrow) between the distal pigment cells in all three species (A, C, D, F). Abbreviations: 1 = retinula cell; cc = crystalline cone; cgc = corneagenous cells; dpc = distal pigment cells; rc = retinula cell; rh = rhabdom. Modified from [85] based on various sources, with permission of Elsevier.

**Background/comment: **Ommatidia are present in all taxa with compound eyes as defined herein. These include the **lateral eyes **that are often not considered to be compound eyes but to be derived from them found, for example, in Collembola, Zygentoma (e.g., [88]) and Lithobiomorpha (e.g., [90]). The exact components of an ommatidium differ in Xiphosura, Scutigeromorpha and Tetraconata (those representatives of the Tetraconata in which ommatidia are present) (see Figure 23, 24). However, an ommatidium always consists of **rhabdomeric photoreceptor cells **(**retinula**(**r**) **cells**), cornea-secreting epithelial cells (e.g., **corneageneous cells**, see Figure 23) and interommatidial pigment cells. In most mandibulate taxa, crystalline cone-secreting cells are present. In Scutigeromorpha and Hexapoda, the cornea-secreting epithelial cells also contain pigments and are therefore called **primary pigment cells. **There are up to ten of these cells in Scutigeromorpha and two in Hexapoda (e.g., [77,86]). In most Crustacea, two corneageneous cells which do not contain pigment granula produce the **cornea**, which is a purely cuticular structure. In Branchiura and Cirripedia, two pigment-bearing cells are present in the position of the corneageneous cells of other crustaceans. The exact homology relationships between these cells and corneageneous cells and/or primary pigment cells remain unclear because in Ostracoda, two corneageneous cells are present in addition to the aforementioned **pigment cells **(see the discussion in [85]). Interommatidial pigment cells are present in mandibulate and xiphosuran compound eyes. It has been suggested that they are homologous within Mandibulata, but their homology has been questioned between mandibulates and xiphosurans [77]. Additional types of pigment cell might be present (e.g., [258]). A central component of ommatidia in mandibulates is the crystalline cone, which forms as an intracellular secretion product. Functionally, the crystalline cone is part of a **dioptric apparatus **(together with the cornea) that is used for light refraction or reflection. The crystalline cone is made up of four cells in most hexapods, in scutigeromorphs and in many crustacean taxa. However, cones made up of two, three or five cone cells are also present in certain crustacean taxa (Figure 24; see [84,85]). In Xiphosura, a crystalline cone is absent. The retinula cells form a **rhabdom **which in most cases is fused but which might also be open (e.g., [86]). The rhabdom might be a one-layer structure (e.g., in Branchiura, Ostracoda, Anostraca), a two-layer structure (e.g., in Scutigeromorpha) or a multiple-layer structure (Malacostraca, Archaeognatha; see [77] for original references), and is termed simple, bilayered or banded rhabdom, accordingly. The number of retinula cells varies (up to 22 in certain beetles: [259]) but eight are often found in hexapods and crustaceans and this number is considered to be a ground pattern character of Tetraconata.

The subunits of the eyes of other invertebrates differ from those of arthropods [91-93]. In sabellid polychates, for instance, each subunit consists of a single **ciliary photoreceptor cell **[93,260,261]. **Lenses **are formed either by an additional cell or by the cuticle lying in a follicle-shaped depression. The photoreceptor cells do not contain shading pigment, which is located in the pigment cells separating the individual subunits.

### **{33} Orthogon**

An orthogon is a cluster of **➞neurons**. It is part of a **➞nervous system **and consists of at least two pairs of longitudinal **➞neurite bundles**, which are connected at regular intervals by transverse neurite bundles running at right angles to them (i.e., orthogonally). The transverse bundles may form a closed circle (circular bundles or **ring commissures**), or at least connect all the longitudinal bundles present. The thickness of the longitudinal neurite bundles can vary, usually with the ventral one being thicker than the others. An orthogon is not differentiated into **➞ganglia **linked by **➞connectives**.

**Discouraged terms**: none

**Figure 25:**
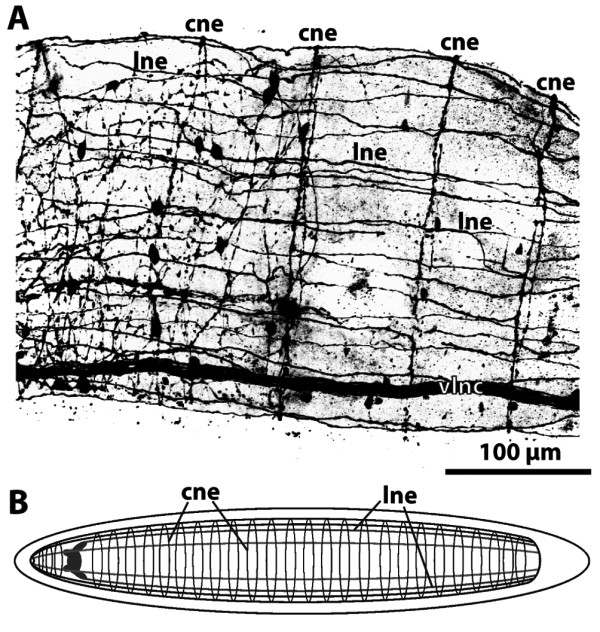
**Orthogon**. **A. **Longitudinal and circular neurites (or neurite bundles) form an orthogon in the trunk of *Tubiluchus troglodytes *(Priapulida). The ventral neurite bundle is distinctly thicker and therefore called ventral longitudinal nerve cord. [Serotonin-like immunoreactivity.] **B. **Schematic representation of the orthogon. Abbreviations: cne = circular neurites; lne = longitudinal neurites; vlnc = ventral longitudinal nerve cord. A: Original: Schmidt-Rhaesa; B: From [222], with permission of Oxford University Press.

**Background/comment**: The term orthogon (Figure 25) was introduced by Reisinger [262] in relation to the architecture of the **➞nervous system **in the flatworm *Bothrioplana semperi *(Bothrioplanida, Seriata, Platyhelminthes), which is composed of four pairs of longitudinal neurite bundles and numerous circular neurite bundles (ring commissures) in a serial arrangement. Reisinger [262] noted: "In summary, we conclude that the nervous system of Turbellaria and consequently that of all Platyhelminthes can be deduced from a simple, geometrical ground pattern, a netlike, right-angled plexus, the orthogon." (p. 146, translated from German). This concept was soon popularized by Hanström [54], who argued that an orthogon of this nature played a key role in nervous system evolution, in particular in the transition from a **➞plexus **as present in diploblastic animals to the concentrated nervous system in Bilateria (Figure 18). Without using these words, Hanström thus proceeded on the assumption that the orthogon was a ground pattern character of Bilateria. Many years later, Reisinger [263] concluded that the orthogon can be regarded as an ancestral character of the Spiralia. By stating that the nervous systems in Deuterostomia cannot be derived from an orthogon, he implicitly called into question Hanström's [54] hypothesis that the orthogon was a character of the bilaterian ancestor. Orthogonal arrangements of longitudinal neurite bundles connected by transverse bundles are abundant among protostome taxa (see, e.g., [222] for a summary). They can differ, however, in several aspects. The number of longitudinal and circular elements can differ considerably, and the thickness of the longitudinal bundles can vary. The transverse neurite bundles often only connect the longitudinal bundles ventrally. For the purposes of the definition it is appropriate to impose a minimum requirement that all the longitudinal neurite bundles present are connected over the ventral midline by transverse elements which must be serially arranged, but the minimum number of the latter is never stated. As the minimum requirement for seriality is two, two transverse bundles may be regarded as the minimal complement of an orthogon.

### **{34} Photoreceptor cell**

A photoreceptor cell is a **➞receptor cell**. It is part of a **➞sensory organ**. It contains photosensitive pigments. The adequate stimulus is light.

**Discouraged terms:** photoreceptor, photosensitive cell

**Figure 26:**
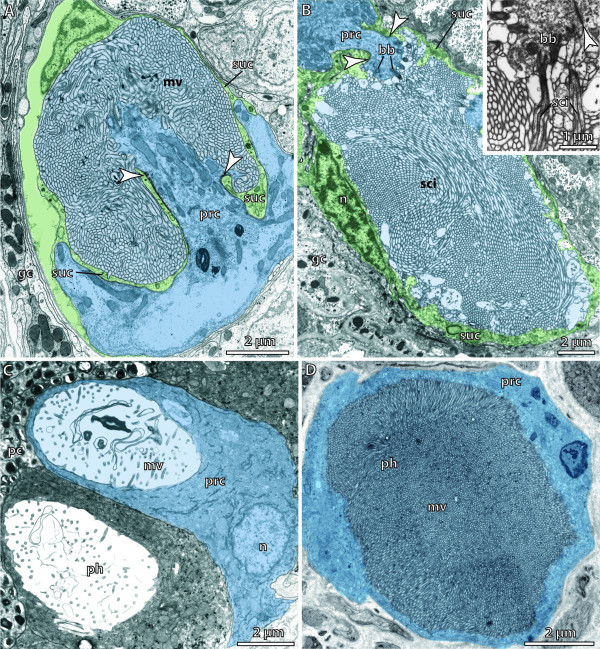
**Types of photoreceptor cells and extraocular ocelli**. Photoreceptor cells and their rhabdomeric microvilli or cilia are labelled in blue and supportive cells in green. **A. ***Microphthalmus similis *(Annelida). Rhabdomeric photoreceptor cell forming an extraocular ocellus with thin supportive cell. Arrowheads point to junctional complexes. [TEM micrograph. Manually labelled.] **B**. *Pisione remota *(Annelida). Ciliary photoreceptor cell and supportive cell. Arrowheads point to junctional complexes. Inset: Enlargement of photoreceptor cell apex with basal bodies and sensory cilia. [TEM micrograph.] **C, D**. Phaosomes of clitellates. **C**. *Stylaria lacustris *(Naididae). Two phaosomous photoreceptor cells of pigmented eye. Note the low density of sensory microvilli. [TEM micrograph.] **D**. *Helobdella robusta *(Euhirudinea, Rynchobdelliformes). Extraocular phaosomous photoreceptor cell (blue). [TEM micrograph.] Abbreviations: bb = basal body; gc = glial cell; mv = sensory microvilli; n = nucleus; pc = pigment cell; ph = phaosome; prc = photoreceptor cell; sci = sensory cilium; suc = supportive cell. Originals: A, B, C: G. Purschke; D: J. Gosda.

**Background/comment: **In the literature, the term photoreceptor is not used unambiguously and may refer to a number of different structures [103,222]. Photoreceptor cells exhibit great structural variability but as a basic principle, the photopigment-bearing structures or organelles are part of the apical plasma membrane domain, the surface of which is typically found to be enlarged in order to provide more space to accommodate the photosensitive pigment (Figure 7, 8, 26B). According to the type of photopigment-bearing structure, a general distinction is made between ciliary and rhabdomeric photoreceptor cells. A **ciliary photoreceptor cell **(Figure 26B) uses ciliary membranes to house the photosensitive pigment. The photosensitive surface area may be optimised by cilia equipped with numerous branches (which may be similar to microvilli in appearance; Figure 26B) or by a high number of cilia per cell. It is well known that ciliary photoreceptor cells occur in vertebrates, but they are present in numerous invertebrates as well, though not usually associated with **pigment cells **in this case and thus frequently described as photoreceptor-like structures. Experimental evidence of **photoreception **in photoreceptor-like structures has only been presented for *Platynereis dumerilii *[124] so far. In a **rhabdomeric photoreceptor cell **(Figure 26A), the light sensitive parts are represented by microvilli which are often highly ordered and densely packed. A cilium, or vestiges thereof, are often present and may project into the ocellar cavity among the phalanx of sensory microvilli. The function of such **accessory cilium **is unknown [95]. The light-sensitive molecules associated with the membranes of the cilia and microvilli are rhodopsins formed by the carotinoid retinal and the protein opsin. Opsins diversified into different types very early in metazoan evolution, leading to opsin families. A number of different ciliary and rhabdomeric opsins are recognized today [104,106,107,122,124,264,265]. Each forms a distinct family, which indicates that ciliary and rhabdomeric photoreceptor cells differentiated very early in metazoan evolution. The split most likely occurred at the base of Bilateria. In most cases, photoreceptor cells are devoid of shading pigment, which is housed in **pigmented supportive cells**. However, there are several examples of photoreceptor cells containing shading pigment (see above **➞eyes**).

Irrespective of receptor cell type, the photopigment-bearing structures typically project into an extracellular space which is either formed by the receptor cells alone or by **➞receptor cells**, **supportive cells **and **cornea **cells [94,95,106,107]. In typical invertebrate eyes, the photoreceptor cells are part of an epithelium called the **retina **and are attached to their neighbours by typical junctional complexes: a **zonula adhaerens **followed by a **septate junction**. Well-known are the photoreceptor cells which occur in pigmented eyes, though **extraocular photoreceptors**, also known as **unpigmented ocelli**, which are not situated within an eye, are also widely distributed among invertebrates. Usually, individuals of a single species bear more than one type of photoreceptive structure which, as a rule, employ different types of receptor cell. Extraocular photoreceptor units are usually small and rarely comprise more than two cells: a photoreceptor cell and an unpigmented supportive cell (Figure 26A, B). due to the fact that Because they are hard to find in larger animals, requiring electron microscopy and serial sectioning to detect them, our knowledge of these structures is patchy. Unpigmented eyes may thus erroneously have been described as phaosomes in the past (see below) and, as a consequence, data from the literature must be treated with care.

A **phaosome **is a third type of photoreceptor cell that is not associated with supportive cells. The photoreceptive processes are housed in a seemingly intracellular vacuole, the phaosome, which arises through the invagination and closure of the apical cell membrane (Figure 26C, D). With exception of clitellate annelids, where they are widely distributed and form the only photoreceptor cell present [95,266], phaosomes only occur sporadically in metazoans. They are usually extraocular and occur in various places, though leeches and certain naidid oligochaetes possess phaosomal eyes [266]. Both microvilli and cilia may be present. The terms 'phaosome' and 'extraocular photoreceptor' have often been used interchangeably due to an incorrect application of the definition or the unrecognized presence of a supportive cell. Formerly regarded as a primitive type of photoreceptor cell [267,268], the evidence is increasing that they may actually be highly derived structures which, at least in clitellate annelids, most likely evolved from rhabdomeric photoreceptor cells ([95], Döring et al. unpublished observation). All phaosomous photoreceptor cells may thus turn out to be a subtype of one of the two receptor cells mentioned above.

### **{35} Pioneer neuron**

A pioneer neuron is a **➞neuron**. It is part of a developing **➞nervous system**. It appears early in development, often exists transiently, and is involved in setting up the scaffold of the developing nervous system. The **➞neurites **of the pioneer neurons serve as pathways for the neurites of neurons, which develop later.

**Discouraged terms: **none

**Figure 27:**
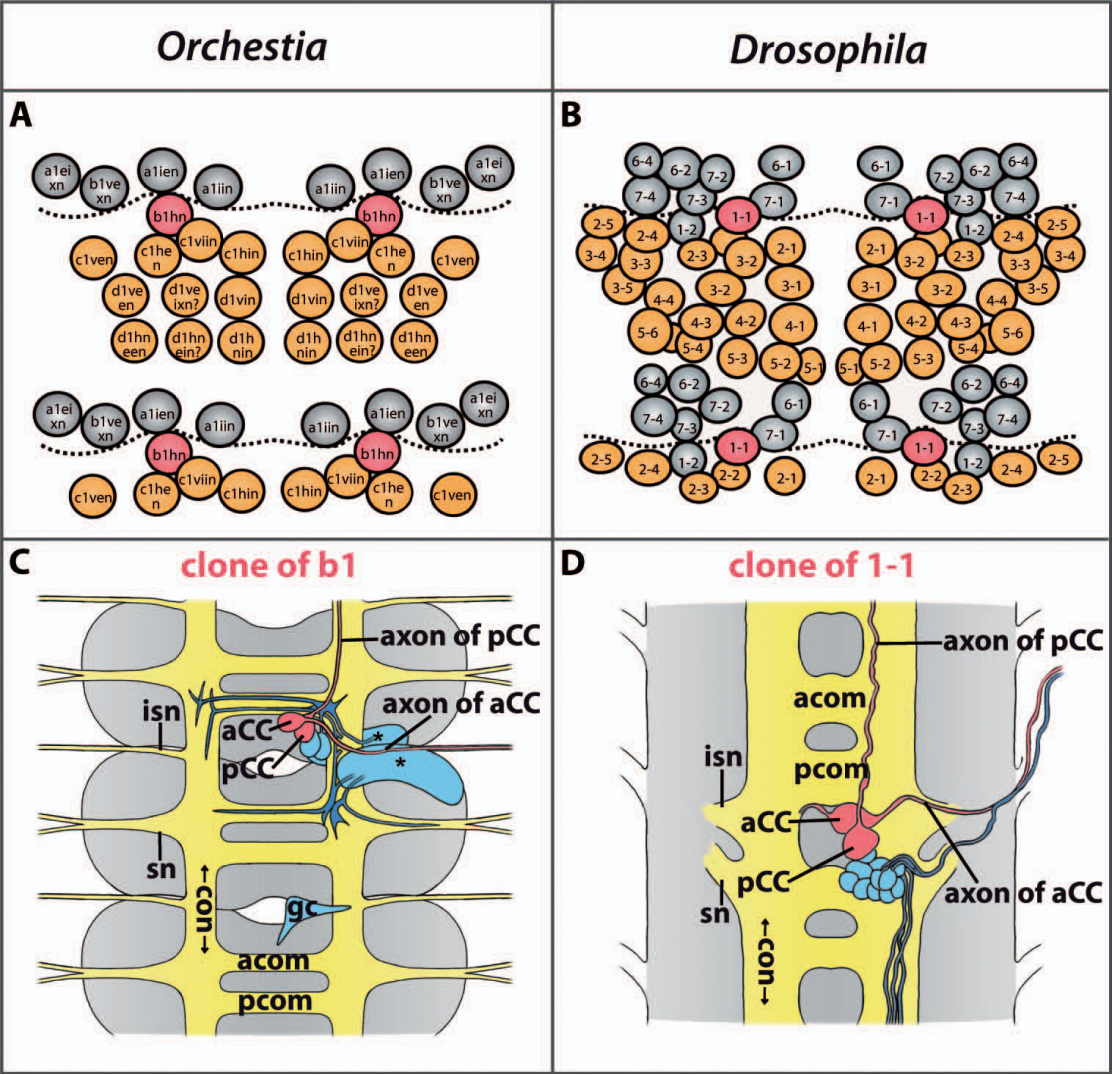
**A comparison of the arrangement of neuroblasts and pioneer neurons between the malacostracan crustacean *Orchestia cavimana *(A, C) and the insect *Drosophila melanogaster *(B, D)**. **A, B**. Scheme of the map of individually identified lateral neuroblasts in both species (midline omitted). Dotted lines mark the segment boundaries. Gray-shaded neuroblasts are engrailed-positive. **C, D**. Schematic representation of the position and axon pathways of the clones of the homologous neuroblasts b1hn and 1-1 in *Orchestia *and *Drosophila*, respectively. The pioneer neurons aCC and pCC (pink) are considered homologous between the two species. Blue neurons and glial cells represent the remaining cells of the respective neuroblast lineage. [Dorsal view.] Abbreviations: 1-1 etc. = labels of individually identified neuroblasts in *Drosophila*; a1eixn etc. = labels of individually identified neuroblasts in *Orchestia*; aCC = pioneer neuron (anterior corner cell); acom = anterior commissure; con = connective; gc = glial cell; isn = intersegmental nerve; pCC = pioneer neuron (posterior corner cell); pcom = posterior commissure. sn = segmental nerve. A, B, C: Modified from [142]; D: Modified from [333]. A, B, C, D: With permission of the Royal Society in London.

**Background/comment: **The function of pioneer neurons has been described in Hexapoda [269-271]. They occur in a specific pattern which finds a correspondence in malacostracan crustaceans (Figure 27; [142,272-274]), but not in Myriapoda [275]. Nothing is known in this respect about other arthropods. If **➞neuroblasts **are present, pioneer neurons are formed by the first **➞ganglion mother cells **budded off by a number of neuroblasts (Figure 17).

### **{36} Plexus**

A plexus is a cluster of **➞neurons**. It is part of a **➞nervous system **and consists of **➞neurites **or **➞neurite bundles **that are arranged in a planar reticular pattern. The **somata **of the **➞neurons **are considered to be part of the plexus.

**Discouraged terms:** epithelial nervous system, diffuse nervous system, nerve net

**Figure 28:**
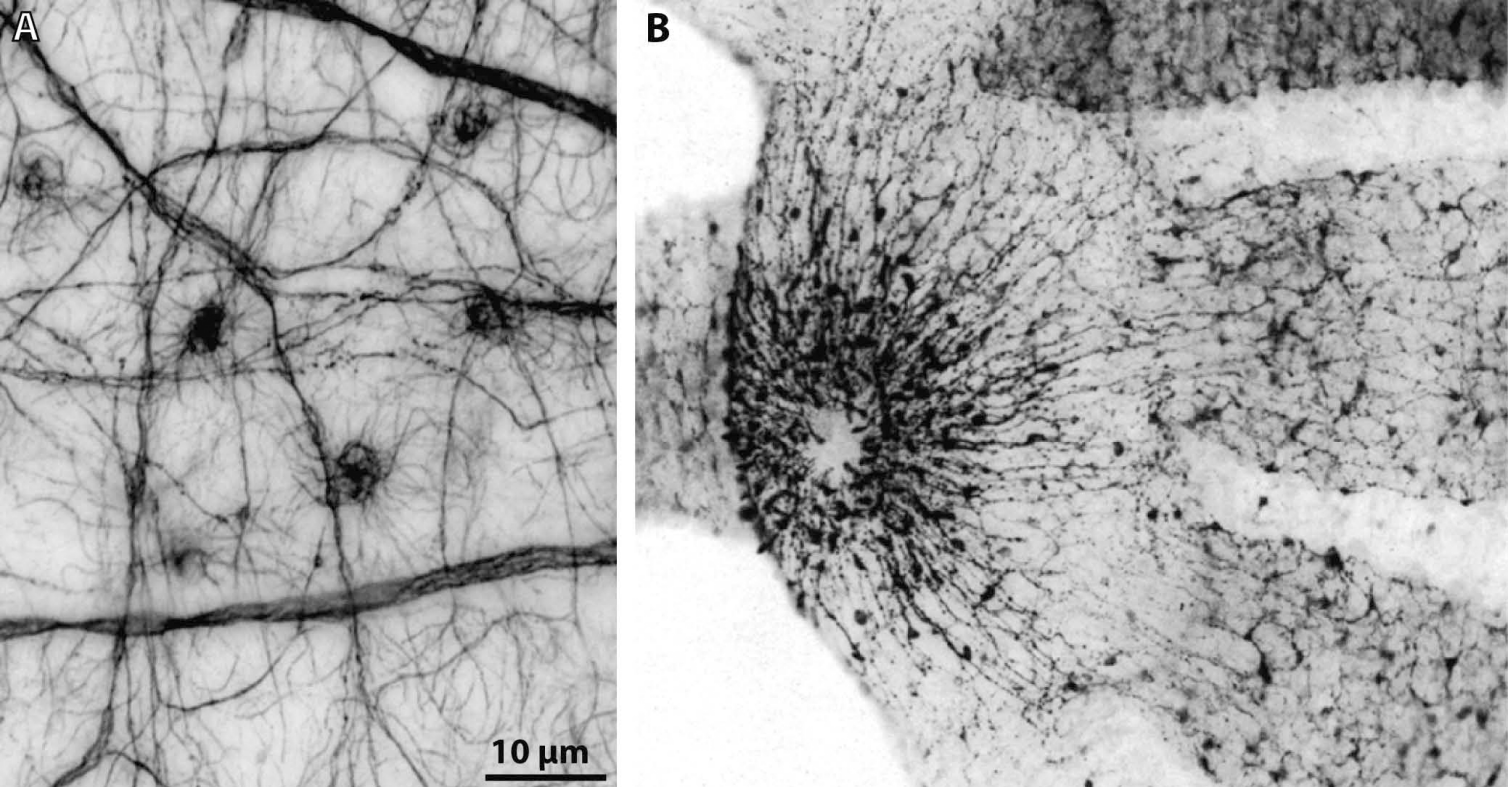
**Plexus**. **A**. Intraepidermal plexus of the chaetognath *Sagitta setosa*. [Acetylated α-tubulin immunohistochemistry.] **B**. Strong agglomeration of sensory cells around the mouth opening of *Hydra attenuata *(Hydrozoa). Radially orientated processes are present in the apical half of the hypostome. A weaker innervation is shown in the tentacles and the upper gastric region. [Whole-mount staining showing RF-amide-like immunoreactivity.] A: From [334], creative common license of BMC; B: From [335], with permission of Springer.

**Figure 29:**
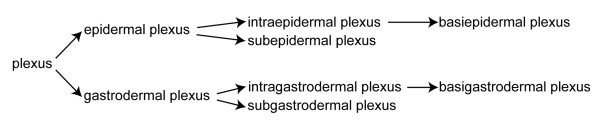
**Terminology of the plexus depending on its relationship to an epithelium**.

**Background/comment: **The entire **➞nervous system **of an animal may be organized as a plexus, as may a specific part thereof (Figure 28). A plexus may or may not contain loosely distributed somata of **interneurons **and **motoneurons **which may be **uni-, bi- or multipolar **(Figure 19). It may or may not contain **➞receptor cells**. It may or may not exhibit **➞synapses **between the receptor cells, motoneurons and interneurons. The plexus is organised in a visibly different way than the **➞orthogon **(Figure 25). A plexus is often associated with an epithelium. The term **epidermal plexus **indicates that an epithelial nervous system is located in the epidermis, whereas **gastrodermal plexus **is associated with the gastrodermis. The spatial relationship between the plexus and the epithelium can be qualified further (Figure 29): In an **intraepidermal plexus**, the neurites are located between the epidermal cells. A **basiepidermal plexus **is a specific subtype of intraepidermal plexus in which the neurites are located between the basal regions of epidermal cells and may have contact with the ECM/basal lamina underlying the epidermis. A **subepidermal plexus **is located below the ECM/basal lamina. The same distinctions can be made for a gastrodermal plexus.

An intraepidermal plexus may function as a **nerve net**. In an intraepidermal plexus, dispersed neurons are connected either by synaptic contact or fusion in such a way as to permit the diffuse conduction of excitation [18]. The term nerve net thus implies a functionally semiautonomous plexus that mediates responses (sensory-motor integration) and must therefore include receptor cells, interneurons and motoneurons that communicate, e.g., via **chemical synapses **or **electrical synapses**. The nervous system of Cnidaria and Ctenophora is typically organized as a plexus which functions as a nerve net [18,190,220,276,277]. An intraepidermal plexus is also a prominent feature of many basal deuterostomes [248,278,279]. It is present in Enteropneusta and Tunicata, and in the basal chordate Branchiostoma, among other taxa. An extensive intraepidermal plexus also characterizes many Protostomia [276], including Annelida [52,56]. Onychophora have recently been shown to have a prominent subepidermal plexus of serotonin-like immunoreactive neurites or neurite bundles [163] which is not obvious in Euarthropoda. Serotonin-like immunoreactive somata are not present in the onychophoran subepidermal plexus.

### **{37} Protocerebral bridge**

The protocerebral bridge is a **➞neuropil**. It is part of the **➞central complex**. Within the protocerebral bridge, **➞neurites **of columnar **➞neurons **form their first collaterals before entering the **➞central body**. The **somata **of these neurons lie adjacent to and dorsal of the protocerebral bridge.

**Discouraged terms**: none

**Background/comment: **The protocerebral bridge can occur as an **➞unpaired midline neuropil **(most Insecta, Figure 4A, B) or be split at the midline of the **➞brain **(e.g., in the malacostracan *Spelaeogriphus lepidops*, Figure 4C). Comparative anatomical studies and behavioural observations on no-bridge *Drosophila *mutants suggest that the protocerebral bridge plays a vital role in coordinating heterolaterally independent leg movements [64,74,280].

### **{38} Receptor cell**

A receptor cell is a **➞neuron**. It is part of the **➞nervous system**. In a signal transduction chain, it is the first neuron that converts an adequate stimulus into an electric signal.

**Discouraged terms:** sensory neuron, sensory cell, receptor

**Background/comment: **The term **receptor **is used in different ways and may apply to a **➞sensory organ**, a receptor cell, the morphological structure of a cell that receives stimulus, or the membrane-bound protein responsible for the first step of signal transduction. There are several types of receptor cell in metazoans. Receptor cells are usually **bipolar cells**, the distal process of which is usually part of an epithelium, mostly the epidermis. Irrespective the position of their **somata**, their dendritic processes bear either cilia and/or microvilli which actually house the proteins responsible for receiving the stimuli. Ciliary receptor cells are the most common and are likely to be involved in almost every kind of sensory perception. Bipolar receptor cells whose somata are located within the epidermis have a short dendritic process and are thus mostly bottle-shaped, giving rise to the name **flask-shaped receptor cells **(see, for example, [113]). Another type of receptor cell is the **free nerve ending**, which ramifies in the periphery and terminates distally as a typical **dendrite **similar to those of **uni- **or **bipolar neurons **([18,222]; Figure 19A, B). They may be connected to **sensory epithelial cells **(**secondary sensory cells, non-neuronal sensory cells**). Although primarily known to occur in Vertebrata, in the acousticolateralis system for instance, secondary cells are thought to be present in cnidarians too and may thus be phylogenetically old structures (see [222]). Receptor cells may occur as unicellular elements, in clusters or as sensory organs of varying degrees of complexity comprising receptor cells and various accessory cells.

There is a great structural variety among the bipolar receptor cells with regard to the size and position of their distal processes, their somata and their **axons**. The same applies to the number and structure of cilia and microvilli, which may be uni- or multiciliated and their axonemes often modified (e.g., [94]). In arthropods and other ecdysozoans, the bipolar receptor cells are connected to specialized cuticular differentiations [222,281-283]. The **collar receptor cell **is often regarded as the most primitive receptor cell type. It occurs in almost every aquatic invertebrate taxon and might have evolved already in early eumetazoans (see [222,284], but see [285]). Its structure somewhat resembles that of the choanocytes of sponges: it is characterized by a stiff cilium and a collar of eight to ten strong microvilli, with eight regarded as the plesiomorphic number [222].

With regard to function, a distinction can be made between **mechanoreception, chemoreception, photoreception, thermoreception, hygroreception, electroreception **and **magnetoreception**. Within invertebrates, arthropods are the best studied group and the function of their receptor cells is often resolved, whereas in many other invertebrate taxa direct experimental evidence is still lacking. As a rule, receptor cells exhibit a distinct morphology which is believed to be correlated to their function. **➞Photoreceptor cells**, for example, are characterized by a significantly larger apical membrane surface, the area which houses the light-sensitive photopigments. This improves photon detection and thus increases sensitivity. Because, in many invertebrates, experimental evidence for the function of a given receptor cell is lacking, function is generally inferred from the fine structure of the cell in question and of the associated stimulus-guiding structures.

### **{39} Rope-ladder-like nervous system**

A rope-ladder-like nervous system is a **➞nerve cord**. It is part of a **➞nervous system **and consists of a series of **➞ganglia **joined by **➞commissures **and **➞connectives**. The ganglia are arranged in an anterior-posterior sequence. The bilaterally arranged pairs of ganglia are transversally joined by at least one commissure. Longitudinally, the ganglia are joined by exactly one connective per side. Segmental **➞nerves **exit the rope-ladder-like nervous system.

**Discouraged terms:** ladder-like nervous system

**Background/comment: **The terms rope-ladder-like nervous system and ladder-like nervous system have been traditionally used to describe the ventral part of the **➞nervous system **in Arthropoda and Annelida (Figure 9, 18). There are several representatives of both taxa, which do not have a rope-ladder-like nervous system according to the definition above, including Onychophora (these do not have ganglia; [162]) and various annelids, in particular oligochaetes [52].

The rope-ladder-like nervous system is embedded into the remaining nervous system and can be complemented by additional elements such as longitudinal **➞neurite bundles**. In polychaets, a ventral median neurite bundle is often present, for example [52]. The polychaete *Dinophilus gyrociliatus *(Dinophilidae) has a rope-ladder-like nervous system, which is connected to further longitudinal and ring-like neurite bundles, which are arranged orthogonally [286]. This phenomenon is widespread in annelids [52]. Interestingly enough, additional median and lateral neurite bundles and ring-like structures are present in the crustacean *Derocheilocaris remanei *(Mystacocarida; [73]).

### **{40} Sensory organ**

A sensory organ is a cluster of **➞cells**. It is part of a **➞nervous system **and consists of receptor cells, which form a multicellular unit that may include accessory cells, which serve as supportive structures, stimulus-guiding structures or protective structures.

**Discouraged terms:** sense organ

**Background/comment: **Sensory organs are those structures, which perceive sensory stimuli and transform them into electric signals recognizable by the **➞nervous system **or directly by the **effector **cells. Simple systems in which the receptor cells are directly connected to cells equipped with motile cilia are present in many invertebrate larvae, for instance [113]. Simple sensory organs may consist of nothing more than a cluster of receptor cells, but as a rule, a sensory organ comprises accessory cells, which serve as supportive structures, stimulus-guiding structures or protective structures for the receptor cells. The result are complex structures which often only admit sensory stimuli from a certain direction, thus conveying information not just about the nature of a stimulus, but also about its intensity and direction [18,222]. For the modes of stimulus that are generally distinguished, see **➞receptor cell**.

With the exception of Porifera and Placozoa, sensory organs of varying degrees of complexity occur in almost every higher metazoan group. The most ubiquitously distributed sensory organs are probably **➞eyes**, while systems such as statocysts, auditory organs and olfactory organs appear to be restricted to more limited groups of taxa. Even Cnidaria may possess highly developed sensory organs, as illustrated by the **rhopalia **observed in scyphozoan and cubozoan medusae. These intricate sensory organs reach the highest level of complexity in cubomedusae [284], where they consist of an (endodermal!) statocyst, two **lens **eyes and two simple eye pits, all of which receive stimuli from different directions (e.g., [114]). These sensory structures are associated with conspicuous epidermal neuronal condensations at the base of the rhopalia (see **➞brain**).

### **{41} Synapse**

A synapse is a cell-to-cell junction. It is part of the **➞nervous system **and consists of pre- and postsynaptic components. It is situated between a **➞neuron **and another cell (e.g., neuron, muscle cell, gland cell) and mediates the transduction of an electric signal between them.

**Discouraged terms**: none

**Background/comment**: This term is discussed at length by Bullock and Horridge [18] and we adhere to their rather broad definition. These authors also discuss functional concepts of the synapse and the historical aspects of these concepts. In **electrical synapses**, current from the presynaptic membrane is sufficient to excite the postsynaptic membrane. In **chemical synapses**, the postsynaptic membrane is only excited by **➞neuroactive substances **packed in vesicles that translocate from the pre- to the postsynaptic side across the synaptic cleft. Chemical synapses thus represent a derived form of paracrine signaling [223]. Most chemical synapses only permit uni-directional information transfer. Invertebrates display a greater complexity of postsynaptic organization than vertebrates in that the presynaptic release site approximates multiple postsynaptic elements to form dyad, triad or tetrad sites [185,287]. The evolutionary origin of synapses and the gradual acquisition of the molecular tool kit to form synapses are not well understood but are the topic of ongoing research using comparative proteomics and genomics (e.g., [176,288]; review by [223]). Because synapses can be viewed as highly specialized paracrine information transmission systems which may have evolved gradually and continuously [223], there is an inherent problem in deciding what constitutes a synapse in some taxa. Sponges, for example, which lack neurons and, therefore, clearly recognizable synapses, nevertheless possess a nearly complete set of post-synaptic protein homologs which indicate that a remarkable level of protein complexity was present at the origin of Metazoa, possibly predating nervous systems [176].

### **{42} Syncerebrum**

A syncerebrum is a **➞brain**. It is part of a **➞nervous system**. It is formed by the fusion or close association of several **➞neuromeres**.

**Discouraged terms: **none

**Background/comment: **Interpretation of the arthropod syncerebrum is very theory-laden, biased by concepts combining ideas on phylogenetic relationships and the nature and origin of segments and segmentation with embryological and functional considerations (see [13]). As a result, numerous contradictory hypotheses about head and brain composition in arthropods have been put forward and continue to be formed (see [289-291] for summaries and discussion of older views, and [292-294] for recent discussion).

The syncerebrum of arthropods is understood as being the result of cephalization, i.e., the structural and functional transformation of segmental (postoral) trunk **➞ganglia**, which are more or less fused to the preoral ancestral brain. However, the structural characterization of the subunits constituting the arthropod brain is somewhat problematic, since neither in the adult brain nor during development are unambiguous boundaries or specific characteristics which would make it possible to identify such subunits recognizable. The expression patterns of segment polarity genes (e.g., engrailed, wingless) have been proven to be helpful in this respect because they more or less resolve the number and spatial arrangement of the morphological units involved in head and brain formation (although this still leaves room for interpretation concerning the evolutionary origin and genealogical relationships of these structures).

The euarthropod syncerebrum is now widely assumed to be tripartite, consisting of a **protocerebrum**, **deutocerebrum**, and **tritocerebum**. During development, the anlagen of these subunits are aligned along the antero-posterior body axis and exhibit, at least transiently, a specific circumesophageal arrangement (Crustacea: [71,295]; Hexapoda: [296-300]; Xiphosura: [301,302]; Pycnogonida: [159]). However, during development, the arrangement of the subunits of the brain in relation to the body axis can be altered, resulting, for instance, in a postero-dorsal protocerebrum [1]. Moreover, rearrangements and fusion processes often mean that the circumesophageal sequence of the subunits of the brain is concealed in the adult brain. Each of the serially arranged brain subunits can in itself be compartmentalized to some degree. This is particularly true of the protocerebum and deutocerebum, which are often subdivided into a number of structurally and developmentally definable subparts formed by clusters of **➞neurons **and distinct **➞neuropil **regions (e.g., [1,66,303]). Some prominent examples of these are ➞**unpaired midline neuropils **(Figure 4), ➞**mushroom bodies **(Figure 12) and **➞olfactory glomeruli **(Figure 22).

The **protocerebrum **receives input from the lateral **➞compound eyes **and the **➞median eyes**, if present (Figure 13). It contributes **➞neurites **at least to the preoral **➞commissure(s) **of the brain. Traditionally, the protocerebrum (or part of it, see below) is viewed as the brain part of the non-segmental acron, which means that it corresponds to the ancestral brain of the Bilateria. In contrast to this, the deuto- and tritocerebrum are mostly considered to be derived from the ganglia proper of true segments. Some authors favour a subdivision of the protocerebrum into the **archicerebrum **and the **prosocerebrum**, with the latter being part of the first true segment, the 'pre-antennal segment'. According to this view, only the archicerebrum belongs to the asegmental acron [290]. However, the concept of the acron is based on the view that annelids and arthropods together form the taxon Articulata. In the light of the current evidence in favour of the Ecdysozoa, the acron concept becomes meaningless. Accordingly, some authors interpret the protocerebrum as being derived from a true ganglion of the anteriormost segment (for a review see [292]).

The **deutocerebum **in myriapods, crustaceans and hexapods is associated with the (first) antennae. It was traditionally assumed that the antennae and the corresponding deutocerebrum are reduced in Chelicerata (e.g., [18,289-291,293]). On the basis of the expression of segmentation genes, Hox genes and neurogenetic data, however, the new view is that Chelicerata indeed possess a deutocerebrum, and that it is connected to the chelicerae ([159,301,304,305]; see [292]). While the traditional textbook view suggests that the commissure of the deutocerebum is preoral, recent investigations have revealed a more complicated scenario in which the deutocerebrum encompasses the esophagus with contralaterally projecting anterior and posterior neurons (e.g., [300,301]).

The **tritocerebrum **is associated with the second antennae in Crustacea and the pedipalps in Chelicerata. Myriapods and hexapods lack appendages in this region, which is referred to as the intercalary segment in these taxa. The tritocerebrum arises from postoral ganglion anlagen, which in the majority of cases migrate anteriorly during development. However, in adults, they are subject to varying degrees of cephalization across the different euarthropod groups. While they are clearly fused to the proto- and deutocerebrum in many hexapods, myriapods and crustaceans [18,54,294], they remain separate in a postoral position in some chelicerates and some crustaceans [18,54,70,293,306-308]. This highlights a certain ambiguity with regard to the posterior boundary of the euarthropod syncerebrum. However, even when the tritocerebral ganglia are postoral and/or structurally similar to trunk ganglia, they nevertheless connect to a cephalized appendage such as an antenna and might thus be considered part of the brain. As a general characteristic, the tritocerebral commissure(s) are always postoral, irrespective of the position of the tritocerebral ganglia (e.g., [18,293,294]).

In addition to the syncerebrum, a cephalisation of a number of segments is observed in a variety of euarthropod groups, indicated by feeding or sensory appendages and a fusion of ganglia (subesophageal ganglion). This phenomenon renders the posterior brain boundary even more problematic.

The brain of onychophorans is also considered to be a morphologically composite structure [42,309]. Recent neuroanatomical and gene expression data on onychophorans suggest that although it exhibits some segmental characteristics [42,310], the central nervous system is not formed by a chain of metameric ganglia as in euarthropods [162,163]. Mayer and Harzsch [162,163] consider this absence of ganglia as the plesiomorphic state within Arthropoda. If this is true, the brain of Onychophora is formed by the fusion of non-ganglionized metameric neuroanatomical units and is thus not a **compound brain **in the strict sense. Moreover, it would suggest that the cephalization of segmental units evolutionarily preceded the formation of proper ganglia in the lineage leading to euarthropods. This could explain the apparent absence of proper ganglia in the euarthropod syncerebrum. Nevertheless, the composite nature of the onychophoran brain qualifies it as a syncerebrum comparable to that in euarthropods. The onychophoran protocerebrum connects to the "antennae" and the lateral eyes, the deutocerebrum is associated with the jaws, and the postoral tritocerebum is the brain part innervating the cephalized appendage of the slime papilla.

The brain of Tardigrada is sometimes interpreted as being tripartite (see [311]). However, not only is the evidence for this view ambiguous, but recent analyses rather suggest that the brain consists of just one part, forming a unit that would thus correspond to the protocerebrum in Onychophora and Euarthropoda (see [311]).

### **{43} Tetraneurion**

A tetraneurion is a cluster of **➞neurons**. It is part of a **➞nervous system **and consists of two prominent pairs of longitudinal **➞neurite bundles**: one inner, ventral pair and one more dorsally situated lateral pair. It may include **➞ganglia**.

**Discouraged terms:** tetraneural nervous system

**Figure 30:**
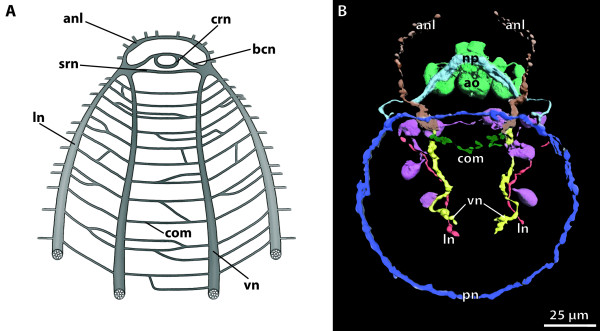
**Tetraneurion**. **A. **Semischematic representation of the nervous system of basal mollusks. Ventral view. Note the different planes in which the ventral and the dorsal nerve chords are situated. **B**. 3D-reconstruction of the nervous system of the creeping-type larva of the entoproct *Loxosomella murmanica*. Somata are depicted in magenta. [3D-reconstruction based on serotonin-like immunoreactivity.] Abbreviations: anl = anterior neural loop; ao = apical organ; bcn = buccal nerve; com = commissure; crn = circumoral nerve; ln = lateral neurite bundle; np = neuropil; pn = prototroch neurite; srn = subradular nerve; vn = ventral neurite bundle. A: After [336]; B: Modified from [37].

**Background/comment: **In the literature, the occurrence of one pair of ventral (pedal) and one pair of lateral (visceral) longitudinal neurite bundles in an animal's nervous system is generally known as **tetraneury **(Figure 30). The term tetraneurion refers to the actual structure (i.e., the two pairs of neurite bundles), while the more commonly used tetraneury refers to the general arrangement (i.e., the presence of two pairs of neurite bundles). Although they are lacking in basal mollusks, ganglia may be part of a tetraneurion, as exemplified in gastropods or bivalves, for example. A tetraneurion has traditionally been considered a defining character of Mollusca. However, an identical situation is found in the creeping-type larva, the proposed basal larval type of Entoprocta [312]. Accordingly, this neural architecture appears to be phylogenetically informative and constitutes an apomorphy of a clade comprising Mollusca and Entoprocta, the Tetraneuralia [37].

### **{44} Tract**

A tract is a **➞neurite bundle**. It is part of a **➞brain **or of a **➞ganglion **and connects different **➞neuropils **with each other.

**Discouraged terms: **none

**Background/comment**: This term is used for neurite bundles within the brain as well as within the ventral **➞ganglia**. An example from the crustacean brain is the olfactory-globular tract which links the olfactory lobe in the **deutocerebrum **with the **hemiellipsoid body **in the **protocerebrum **and is composed of the **axons **of olfactory **interneurons **[149,313,314]. A tract may be composed of axonal profiles of similar diameter, or of wide diameter, fast-conducting profiles of giant axons. **➞Synapses **are usually not present in tracts, but exceptions are known [18].

### **{45} Trochal neurite**

A trochal neurite is a **➞neurite**. It is part of a **➞neuron **and underlies a ciliated trochus.

### **{46} Trochal neurite bundle**

A trochal neurite bundle is a **➞neurite bundle**. It is part of a **➞nervous system **and underlies a ciliated trochus.

**Discouraged terms: **prototroch nerve ring, telotroch nerve ring, prototroch nerve, telotroch nerve, trochus nerve

**Figure 31:**
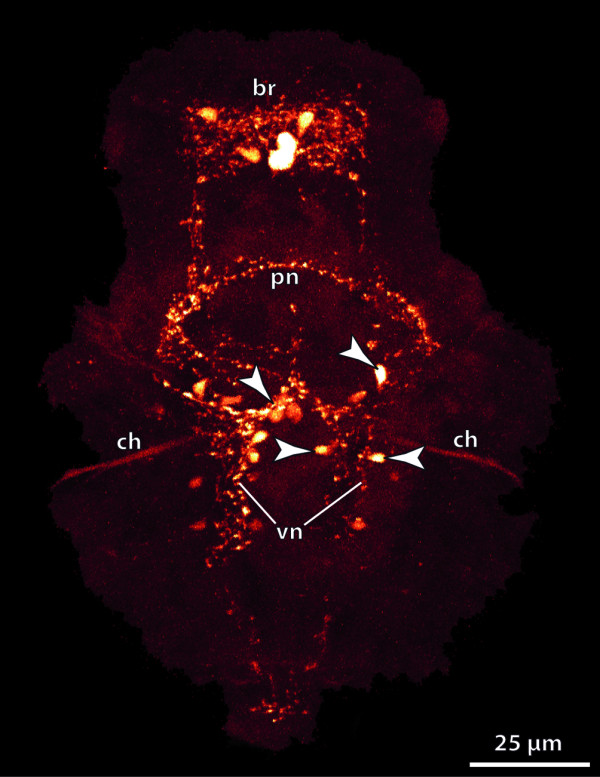
**Nervous system of the trochophore larva of the polychaete annelid *Filograna implexa***. The prototroch neurite, the anlage of the brain, the chaetae, the paired ventral neurite bundle, and the associated somata (arrowheads) are stained. [Serotonin-like immunoreactivity.] Abbreviations: br = brain; ch = chaetae; pn = prototrochal neurite; vn = ventral neurite bundle. Original: A. Wanninger.

**Background/comment: **A trochal neurite bundle or a single **➞trochal neurite **underlies and possibly innervates the ciliated prototroch of most spiralian larvae (Figure 31). Trochal neurites are arranged concentrically to the prototroch, metatroch and telotroch of trochophore larvae, and to homologous structures such as the velum of certain gastropod and bivalve veliger larvae. In addition, trochal neurites may also be associated with the ciliated lobes of the Müller's larva of polyclad platyhelminths, the pilidium larva of nemertines and the ciliated bands of enteropneust and echinoderm larvae [129,133,134,312,315-323]. Many trochal neurites can be detected using antibodies against serotonin. **➞Neurites **underlying ciliary bands have traditionally played an important role in comparative larval neuroanatomy because they have been found in nearly all larval protostomes and deuterostomes. Many late-stage planktotrophic polychaete larvae have longitudinal neurites or neurite bundles (often two) that underlie ventral ciliary bands. These are termed **neurotroch **or **gastrotroch neurites**. Some traditional hypotheses argue that the **ventral ➞nerve cords **of protostomes originated from the fusion of neurotroch neurites, which were thought to have been present in the last common trochophore-like larva of protostomes (e.g., [38]).

### **{47} Unpaired midline neuropil**

 An unpaired midline neuropil is a **➞neuropil**. It is part of a **➞brain **and occurs as an individual neuropil, spanning the midsagittal plane of the brain.

**Discouraged terms**: none

**Background/comment**: The term unpaired midline neuropil is recommended to denominate hitherto unspecified midline neuropils in order to avoid premature homologization between these neuropils and specific unpaired midline neuropils such as the **➞central body**, the **➞protocerebral bridge **(Figure 4) and the **➞arcuate body**. Unspecified midline neuropils have been described in polychaetes [147,254], among other taxa.

## Register of neuroanatomical terms

**Table 1:** Register of neuroanatomical terms.

Left column: All 47 main entries, i.e., those neuroanatomical terms which were given an own definition, are printed in bold. All side entries, i.e., those terms which were not given a specific definition but are as important for neuroanatomical descriptions, are printed in regular. Right column: The numbers refer to all main entries under which the respective term is used. Bold numbers lead to the definition of the respective main entry.	
neuroanatomical term	corresponding main entries {No}
accessory cilium	34

adult eye	9

afferent	15, 19

apical ganglion	1, 10

apical plate	1

apical rosette	1

**apical organ**	**1**, 10

**arcuate body**	**2**, 4, 47

archicerebrum	42

axis cylinder	23

axon	3, 6, 8, 10, 12, 17, 18, 19, 23, 28, 30, 38, 44

basiepidermal plexus	36

biogenic amine	25

bipolar cell	38

bipolar neuron	28,38

**brain**	1, 2, **3**, 9, 10, 12, 13, 14, 17, 18, 19, 22, 26, 37, 40, 42, 44, 47

calyx	17

cell cortex	3, 10, 16, 30

**central body**	2, **4**, 5, 14, 30, 37, 47

**central complex**	4, **5**, 14, 37

central nervous system	10, 19, 21, 42

cerebral commissure	1, 42

cerebral eye	7, 9

cerebral ganglion	3, 10

chemical synapse	36, 41

chemoreception	38

ciliary photoreceptor cell	32, 34

collar receptor cell	38

commissural brain	3

**commissure**	**6**, 10, 39, 42

**compound eye**	**7**, 9, 32, 42

compound brain	3, 42

**connective**	3, **8**, 10, 16, 27, 33, 39

converse eye	9

cornea	9, 32, 34

corneageneous cell	32

cornea-secreting epithelial cell	32

corpora pedunculata	17

crystalline cone	32

cycloneuralian brain	3

dendrite	10, 17, 23, 28, 30, 38

deutocerebrum	31, 42, 44

diffuse nervous system	36

dioptric apparatus	32

effector	19, 40

efferent	19

electrical synapse	36, 41

electroreception	38

epidermal plexus	36

epithelial nervous system	36

everse eye	9, 18

extracerebral eye	9

extraocular photoreceptor	34

extrinsic neuron	28

**eye**	7, **9**, 15, 18, 32, 34, 40, 42

facetted eye	7

flask-shaped receptor cell	1, 38

four-partite eye	15, 18

free nerve ending	38

frontal eye	18

frontal ocellus	15

frontal organ	18

**ganglion**	1, 3, 6, 8, **10**, 12, 14, 16, 19, 20, 21, 22, 26, 27, 28, 31, 33, 39, 42, 43, 44

**ganglion mother cell**	**11**, 26, 35

gastrodermal plexus	36

gastrotroch	46

**glial cell**	3, 10, 11, **12**, 16, 18, 21, 29, 30

**globuli cell**	**13**, 17

hemiellipsoid body	13, 44

hemiganglion	6, 10

hygroreception	38

individually identifiable neuron	28

interneuron	6, 8, 10, 28, 30, 36, 44

interommatidial pigment cell	7, 32

intraepidermal plexus	36

intrinsic neuron	17, 28

inverse eye	9, 18

iris	9

Kenyon cells	13, 17

ladder-like nervous system	39

larval eye	9

**lateral accessory lobe**	4, 5, **14**

lateral eye	9, 15, 32, 42

lateral lobe	14

lateral ocellus	7, 9, 15

lens	9, 32, 40

magnetoreception	38

Markstrang	16

mechanoreception	38

**median eye**	9, **15**, 18, 42

median eye nerve	15

median ocellus	15

**medullary cord**	8, 10, **16**, 20

motoneuron	19, 28, 36

multipolar neuron	10, 28

**mushroom body**	13, **17**, 42

**nauplius eye**	15, **18**

**nerve**	3, 10, 18, **19**, 23

nerve cell	23, 28

**nerve cord**	3, 16, **20**, 22, 24, 39, 46

nerve fiber	23

Nervenfaser	23

nerve net	36

nerve tube	22

**nervous system**	1, 3, 6, 7, 8, 9, 10, 11, 12, 15, 16, 18, 19, 20, **21**, 22, 23, 24, 25, 26, 27, 28, 29, 30, 31, 33, 35, 36, 38, 39, 40, 41, 42, 43, 46

nervous tissue	12, 21, 22, 27

neural canal	22

neural cord	22

**neural tube**	10, 21, **22**

neurenteric canal	22

neurilemma	12

**neurite**	3, 6, 8, 10, 12, 21, **23**, 24, 28, 30, 35, 36, 37, 42, 45, 46

**neurite bundle**	6, 8, 12, 19, 20, 21, **24**, 33, 36, 39, 43, 44, 46

**neuroactive substance**	**25**, 28, 41

**neuroblast**	11, **26**, 29, 35

neuroglia	12

**neuromere**	6, **27**, 42

**neuron**	3, 4, 10, 11, 12, 13, 14, 17, 20, 21, 23, 25, **28**, 29, 31, 33, 35, 36, 37, 38, 41, 42, 43, 45

**neuronal precursor**	11, 26, **29**

neuronal progenitor	29

neuropeptide	25

**neuropil**	2, 3, 4, 5, 6, 7, 10, 13, 14, 15, 16, 17, 28, **30**, 31, 37, 42, 44, 47

neuropore	22

neurotroch	46

neurulation	22

non-neuronal sensory cell	38

ocellus	7, 9, 15

**olfactory glomerulus**	30, **31**, 42

**ommatidium**	7, 9, 15, **32**

**orthogon**	6, **33**, 36

pedunculus	17

perikaryon	28

perilemma	12

perineurium	12

peripheral nervous system	21

phaosome	34

photoreception	9, 34, 38

photoreceptor	9, 15, 19, 34

**photoreceptor cell**	9, 32, **34**, 38

photosensitive cell	34

phototaxis	9

pigment cell	3, 9, 18, 32, 34

pigment-cup eye	9

pigmented supportive cell	9, 34

**pioneer neuron**	26, **35**

**plexus**	23, 33, **36**

primary neurite	10, 23, 28

primary pigment cell	32

prosocerebrum	42

**protocerebral bridge**	4, 5, **37**, 47

protocerebrum	5, 15, 18, 31, 42, 44

prototroch nerve	46

prototroch nerve ring	46

prototype eye	9

pseudounipolar neuron	28

receptor	21, 25, 38

**receptor cell**	1, 16, 19, 34, 36, **38**, 40

retina	9, 34

retinula(r) cell	32

rhabdom	32

rhabdomeric photoreceptor cell	32, 34

rhopalium	3, 9, 40

ring commissure	6, 33

**rope-ladder-like nervous system**	6, 10, **39**

secondary sensory cell	38

sense organ	40

sensory cell	18, 38

sensory epithelial cell	38

sensory neuron	38

**sensory organ**	1, 9, 34, 38, **40**

septate junction	34

sheath	12

soma	3, 8, 10, 13, 16, 17, 19, 23, 27, 28, 30, 36, 37, 38

stemmata	7, 9, 15

subepidermal plexus	36

supportive cell	9, 12, 34

supraesophageal ganglion	3

**synapse**	10, 28, 30, 31, 36, **41**, 44

**syncerebrum**	2, 3, 4, 5, 7, 15, **42**

tapetum	9, 18

telotroch nerve	46

telotroch nerve ring	46

tetraneural nervous system	43

**tetraneurion**	1, **43**

tetraneury	43

thermoreception	38

three-partite eye	18

**tract**	3, 6, 10, 19, 30, **44**

tritocerebrum	3, 42

**trochal neurite**	**45**, 46

**trochal neurite bundle**	45, **46**

trochus nerve	46

unipolar neuron	10, 23, 28

**unpaired midline neuropil**	2, 4, 37, 42, **47**

unpigmented ocellus	9, 34

ventral body	14

ventral nerve cord	20, 46

vitreous body	9

zonula adhaerens	34

## Competing interests

The authors declare that they have no competing interests.

## Authors' contributions

SH, RL, GP, SR, ASR, GS, TS, LV, and AW provided the text of the entries. GB, CD, SF, MF, PG, CMH, SK, OSM, CHGM, VR, BHR, and MEJS discussed with the authors of the entries all aspects of this glossary at various meetings. CMH redrew many of our figures, SK took care of the exact labelling and final layout of figures and MEJS organized the register, references and figure legends. SR and SH organized the final draft. All authors have read and approved the final draft of the manuscript.
